# *Panacis Quinquefolii Radix* Polysaccharides Alleviate Depressive‐Like Behaviors in Chronic Unpredictable Mild Stress‐Induced Mice by Suppressing Complement C1Q/C3‐Mediated Microglial Synaptic Pruning and Modulating Gut Microbiota

**DOI:** 10.1002/cns.70859

**Published:** 2026-03-31

**Authors:** Mengjun Xie, Lingling Feng, Ruyi Li, Mengqi Li, Liang Shen, Minghui Zhang, Yutong Wei, Qiao Yin, Datong Wang, Liqing Chen, Kai Song, Peipei Wang, Qifei Cong

**Affiliations:** ^1^ Jiangsu Key Laboratory of Drug Discovery and Translational Research for Brain Diseases, Institute of Neuroscience Soochow University Suzhou China; ^2^ Department of Nephrology the Second Affiliated Hospital of Soochow University Suzhou China; ^3^ Department of Marine Pharmacology, College of Food Science and Technology Shanghai Ocean University Shanghai China; ^4^ Department of Neurology, Clinical Research Center of Neurological Disease the Second Affiliated Hospital of Soochow University Suzhou China; ^5^ Jiangsu Key Laboratory of Drug Discovery and Translational Research for Brain Diseases, College of Pharmaceutical Sciences Soochow University Suzhou China; ^6^ School of Life Sciences, MOE Key Laboratory of Geriatric Diseases and Immunology, Suzhou Medical College of Soochow University Soochow University Suzhou China; ^7^ Institute of Neurological Diseases Soochow University‐Suzhou Blue Cross Brain Hospital, Soochow University Suzhou China; ^8^ Biomedical Basic Research Center of Jiangsu Soochow University Suzhou China

**Keywords:** chronic unpredictable mild stress, complement, gut microbiota, microglia, *Panacis Quinquefolii Radix*, polysaccharide

## Abstract

**Aims:**

*Panax quinquefolius Radix* (American ginseng) is a medicinal herb used for its neuroprotective and tonic effects. However, the antidepressant potential of its polysaccharide components is not well studied. This research aimed to investigate the antidepressant effects of XYS1, a polysaccharide from American ginseng, focusing on mechanisms related to the complement system and the gut‐brain axis.

**Methods:**

A chronic unpredictable mild stress (CUMS) mouse model was used to induce depressive behaviors. Mice were treated with XYS1 via oral gavage, followed by assessments of behavior, molecular changes, and gut microbiota.

**Results:**

XYS1 treatment significantly alleviated depression‐like behaviors in CUMS mice, as demonstrated by reduced immobility time in the TST and FST, and increased sucrose preference and body weight. Mechanistically, XYS1 attenuated complement system activation by downregulating C1Q expression in microglia and C3 expression in astrocytes, not only in the hippocampal CA1 region but also in the mPFC and PVN, as well as in the colon. Furthermore, XYS1 inhibited microglial activation and associated synaptic phagocytosis, preserved glutamatergic neuron density, restored excitatory synapse density, and reversed CUMS‐induced gut microbiota dysbiosis by enriching Bacillota and reducing Bacteroidota abundance. Additionally, XYS1 effectively mitigated both colonic and systemic inflammation, reducing pro‐inflammatory cytokines TNF‐α and IL‐1β and complement components C1Q and C3, while restoring anti‐inflammatory IL‐10 levels, thereby modulating the gut‐brain axis.

**Conclusions:**

XYS1 exerts antidepressant effects by modulating the C1Q/C3 complement pathway, inhibiting microglial‐mediated synaptic pruning, and restoring gut microbiota homeostasis.

## Graphical abstract

XYS1 ameliorates depressive‐like behaviors in CUMS mice via the gut‐brain axis by inhibiting complement activation, suppressing microglial phagocytosis, restoring gut microbiota, and alleviating inflammation.

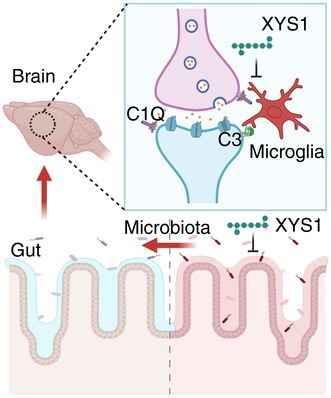

## 1 Introduction

Depression has emerged as a significant global health concern, markedly exacerbated by the COVID‐19 pandemic [1]. The pathogenesis of depression remains incompletely understood. Fundamentally, depression may represent a disorder of synaptic dysfunction [2, 3], suggesting that enhancing synaptic plasticity could be an innovative therapeutic avenue. Current pharmacological interventions predominantly include tricyclic antidepressants (TCAs), monoamine oxidase inhibitors (MAOIs), and selective reuptake inhibitors (SSRIs and SNRIs). Despite their widespread use, these agents are hindered by delayed therapeutic onset and issues with patient adherence. Consequently, there is an urgent need for the development of novel antidepressant drugs that can induce rapid effects with low toxicity profiles and minimal side effects.

*Panacis Quinquefolii Radix* (PQR), the dehydrated root of *Panax quinquefolius* (American ginseng), is a highly esteemed medicinal entity noted for its unique therapeutic properties [4]. Comprehensive chemical analyses have identified a range of active constituents, including saponins, polysaccharides, fatty acids, and flavonoids, as well as essential nutrients in PQR. Similar to Asian ginseng, American ginseng is a versatile herb rich in bioactive constituents, primarily ginsenosides and polysaccharides [5, 6, 7]. Moreover, *Panacis Quinquefolii Radix* polysaccharides possess significant pharmacological functions, including immune regulation, antioxidant, anti‐hyperglycemic, and anti‐inflammatory properties [8, 9, 10, 11, 12, 13, 14, 15, 16, 17, 18]. However, the specific active components and immunomodulatory properties of different purified polysaccharide fractions responsible for these effects remain unidentified.

Recent research has highlighted the complement system's significant role in a variety of conditions affecting both the central and peripheral nervous systems [19, 20, 21, 22], encompassing infectious [23], autoimmune, and neurodegenerative disorders [24]. Additionally, its involvement is increasingly acknowledged in neuropsychiatric conditions, especially depressive disorders. Previous investigations have demonstrated that different stress paradigms, including corticosterone administration, chronic restraint stress, and chronic unpredictable stress, lead to spatially heterogeneous activation of complement pathways within the brain [25]. This consistent pattern of complement activation across various studies underscores its status as a conserved immune response to chronic stressors [26, 27, 28, 29, 30, 31, 32, 33]. However, it remains unclear whether complement‐mediated neuroinflammation contributes to the antidepressant effect of *Panax quinquefolius* polysaccharides.

## 2 Material and Methods

### 2.1 Preparation and Purification of XYS1

Crude polysaccharides were isolated from *Panax quinquefolius Radix* employing a compound enzymatic extraction technique [7]. The powdered radix was first defatted and treated with Papain. The mixture was concentrated and dialyzed, producing freeze‐dried crude polysaccharides called XYS. Fractionation using a DEAE Sepharose Fast Flow column with a NaCl gradient yielded XYS1, the fraction with the highest polysaccharide concentration, eluted at 0.1 M NaCl.

### 2.2 Animal and Ethical Statement

Male C57BL/6J mice, aged 8 weeks, were sourced from Gempharmatech Biotechnology, Nanjing, and kept under specific pathogen‐free conditions at Soochow University. They lived in a controlled environment at 22°C with 40%–60% humidity, on a 12‐h light–dark cycle, with 4–5 mice per cage, separated by sex, and had ad libitum access to food and water. All animal experiments were performed in accordance with the National Institutes of Health Guide for the Care and Use of Laboratory Animals and were approved by the Animal Ethical Committee of Soochow University (Approval No. SUDA20240911A11).

### 2.3 Chronic Unpredictable Mild Stress (CUMS) Protocol

Mice were subjected to various stressors twice daily for 28 days, including a 15‐min tail suspension, 2 h of tube restraint, 30 min on an elevated platform, and ten 2‐s footshocks at 0.7 milliamps. They also experienced 24 h of wet bedding, a cage at a 45‐degree angle, and continuous light exposure. Control mice were handled and group‐housed, while chronically unpredictable mild stressed (CUMS) mice were kept individually to limit social interaction.

### 2.4 XYS1 Treatment Protocol

CUMS modeling mice were systematically allocated to three distinct experimental cohorts: The CUMS group and two additional groups receiving either XYS1 or Fluoxetine (Flu) through gavage. The CUMS+XYS1 group received XYS1 at 500 mg/kg/day, while the CUMS+Flu group received Flu at 15 mg/kg/day. For comparison, both the control group and the CUMS group received normal saline as a vehicle control via gavage.

### 2.5 Behavioral Tests

#### 2.5.1 Tail Suspension Test (TST)

The XR‐XQ202 apparatus (Xinruan Information Technology, Shanghai, China) assessed behavioral responses in mice. After a one‐hour acclimatization in a quiet environment, mice underwent a tail suspension test, with their tails taped 1 cm from the tip to a hook 50 cm above the ground. They were recorded for 6 min, with the last 4 min focused on immobility.

#### 2.5.2 Forced Swimming Test (FST)

The experiment used the XR‐XQ202 forced swim and tail suspension test system. Mice were acclimated in a quiet room for 1 h before being placed in a transparent cylindrical container (30 cm high, 20 cm in diameter) filled with water to a depth of 22 cm at 22°C–24°C. Video recording began when the mice entered the water, focusing on the last four minutes of a six‐minute observation to measure the time spent floating motionless after initial struggle.

#### 2.5.3 Sucrose Preference Test (SPT)

The experimental design involved keeping mice in one cage as follows: (a) Acclimatization with two bottles of 1% sucrose solution from 21:00 on Day 1 to 21:00 on Day 2. (b) On Day 2, one sucrose bottle was replaced with water, and positions were swapped on Day 3 at 09:00. (c) Mice fasted from 21:00 on Day 3 to 21:00 on Day 4 with no water. (d) Testing occurred from 21:00 on Day 4 to 21:00 on Day 5 with one 1% sucrose bottle and one distilled water bottle. Positions were reversed on Day 5 at 09:00, and bottle masses were recorded before and after testing. The sugar water preference index was calculated as follows: Sucrose preference index% = (sugar water consumption/(sugar water consumption + pure water consumption)) x 100%.

### 2.6 CCK‐8 Detection of Cell Viability

BV2 cells were plated at 5 × 10^3^ per well in 96‐well plates and incubated overnight at 37°C. After removing the media, cells were divided into a control group, an LPS‐treated group (1 μg/mL), and four XYS1 treatment groups (0.1, 0.2, 0.5, and 1 mg/mL). After 2 h of XYS1 pre‐treatment, all groups except the control were exposed to LPS for 24 h. Cell viability was assessed using the CCK‐8 assay by adding 10 μL of solution per well, incubating for 2 h, and measuring absorbance at 450 nm.

### 2.7 Western Blot

Cell proteins were lysed with pre‐cooled RIPA buffer containing protease and phosphatase inhibitors. Lysates were centrifuged at 12,000 rpm for 20 min, and protein concentration was measured using a BCA assay. Samples were denatured in loading buffer at 95°C for 5 min, separated by 10% SDS‐PAGE, and transferred to PVDF membranes. After blocking with 5% skim milk for 1 h and washing with TBST, the membranes were incubated overnight with various primary antibodies: Rabbit anti‐CD11b (Abcam, ab133357, 1:1000), mouse anti‐C3 (Proteintech, 66157–1‐ig, 1:1500), rabbit anti‐C1Q (ABclonal, A24519, 1:2000), mouse anti‐Tubulin (Fude Biotechnology, FD0064, 1:2500), rabbit anti‐p‐STAT3 (Abcam, ab32143, 1:5000), rabbit anti‐STAT3 (Cell Signaling Technology, 4904 T, 1:2000), rabbit anti‐p‐P65 (ImmunoWay, YP0191, 1:2000), rabbit anti‐P65 (Cell Signaling Technology, 8242 T, 1:2000), mouse anti‐p‐ERK 1/2 (Santa Cruz Biotechnology, SC‐81492, 1:500), and mouse anti‐ERK 1/2 (Santa Cruz Biotechnology, SC‐514302, 1:500). Following additional washes with TBST, they were incubated with HRP‐conjugated secondary antibodies, Goat anti‐mouse IgG (H + L) HRP (Fude Biotechnology, FDM007, 1:4000) and Goat anti‐rabbit IgG (H + L) HRP (Fude Biotechnology, FDR007, 1:4000) for 2 h. Chemiluminescence detection was performed using an ECL kit, and protein levels were normalized to tubulin, with quantification done using Image J software.

### 2.8 Immunofluorescence Staining

Brain and intestinal tissues from murine models were fixed in 4% paraformaldehyde for 24 h, followed by dehydration in 30% sucrose (in PBS). Samples were sectioned at 30 μm using a cryostat. Tissues were rinsed in PBS and PBST, blocked with 5% sheep serum albumin in PBST, and incubated overnight at 4°C with primary antibodies, including rat anti‐CD68 (Abcam, ab53444, 1:500), guinea pig anti‐VGlut2 (Synaptic Systems, 135404, 1:1000), chicken anti‐Homer1 (Synaptic Systems, 160006, 1:500), mouse anti‐Gephyrin (Synaptic Systems, 147021, 1:500), guinea pig anti‐VGAT (Synaptic Systems, 131004, 1:500), guinea pig anti‐GFAP (Oasis Biofarm, OB‐PGP055, 1:500), rat anti‐C3 (Abcam, ab11862, 1:200), rabbit anti‐C1Q (Abcam, ab182451, 1:1000), rabbit anti‐Glumate (Sigma, G6642, 1:1000), rabbit anti‐GABA (Sigma, A2052, 1:1000), and chicken anti‐IBA1 (Synaptic Systems, 234009, 1:1000). After incubation, tissues were washed with PBST, and secondary antibodies including Goat Anti‐Rat IgG H&L (Thermo Fisher Scientific, A594, 1:1000), Goat Anti‐Guinea Pig IgG (Abcam, ab150185, 1:500), Goat Anti‐Chicken IgY (Abcam, ab150170, 1:1000), Goat Anti‐Mouse IgG1 (Thermo Fisher Scientific, A‐21125, 1:500), Goat Anti‐Rabbit IgG (Abcam, ab150180, 1:500), and Goat Anti‐Rabbit IgG (Abcam, ab150181, 1:1000) were applied. Slides were rinsed, mounted, and imaged using LSM700 and LSM900 fluorescence microscopes.

### 2.9 Histological Analysis

Intestinal tissue specimens were harvested and sectioned into 30 μm thickness using a cryostat. The sections were subsequently mounted on glass slides and stained with hematoxylin and eosin (H&E). The pathological alterations in the intestinal architecture were then analyzed utilizing a VS200 optical microscope.

### 2.10 16S rRNA Analysis of Intestinal Contents

Genomic DNA was extracted from rodent fecal samples using the FastPure Stool DNA Isolation Kit, following the manufacturer's protocol. DNA quality was assessed by 1.0% agarose gel electrophoresis, quantified with a NanoDrop 2000 spectrophotometer, and stored at −80°C. For amplifying the hypervariable V3−V4 region of the bacterial 16S rRNA gene, PCR was performed with primer pairs 338F and 806R on a T100 Thermal Cycler. The PCR products were extracted from a 2% agarose gel and purified with a PCR Clean‐Up Kit. Purified products were quantified using a Qubit 4.0 fluorometer, pooled equimolar, and subjected to paired‐end sequencing on the Illumina Nextseq2000 platform. Bioinformatic analyses of the gut microbiota were conducted using the Majorbio Cloud platform (https://cloud.majorbio.com).

### 2.11 Real‐Time Quantitative Polymerase Chain Reaction

Total RNA was extracted from mouse brain tissues using the FreeZol Reagent kit (Cat. No. R711, Vazyme Biotech Co. Ltd., Nanjing, China). Subsequently, reverse transcription was performed with the HiScript III RT SuperMix for qPCR (+gDNA wiper) kit (Cat. No. R323, Vazyme Biotech Co. Ltd., Nanjing, China) to synthesize complementary DNA (cDNA). qRT‐PCR was carried out on an ABI 7500 Real‐Time PCR System (Thermo Fisher Scientific, Waltham, MA, USA). The relative mRNA expression levels of target genes were calculated using the 2−^ΔΔCt^ method. The qPCR primers were purchased from GENEWIZ. Primers sequences are: *GAPDH* FW: 5′‐TGTGAACGGATTTGGCCGTA‐3′; *GAPDH* RV: 5′‐GGCCTCACCCCATTTGATGT‐3′; *C1q* FW: 5′‐TTCGGCAGAACCCAATGACG‐3′; *C1q* RV: 5′‐TGGTATGGACTCTCCTGGTTG‐3′; *C3* FW: 5′‐TTCTCCGCAGAGTTTGAGGT‐3′; *C3* RV: 5′‐TTCTTATCGCCATCCTGGAC‐3′; *Tnf‐α* FW: 5′‐TAGCCCACGTCGTAGCAAAC‐3′; *Tnf‐α* RV: 5′‐TGTCTTTGAGATCCATGCCGT‐3′; *Il‐1β* FW: 5′‐TGCCACCTTTTGACAGTGATG‐3′;*Il‐1β* RV: 5′‐ATGTGCTGCTGCGAGATTTG‐3′; *Il‐10* FW: 5′‐GCTCTTACTGACTGGCATGAG‐3′; *Il‐10* RV: 5′‐CGCAGCTCTAGGAGCATGTG‐3′.

### 2.12 Enzyme‐Linked Immunosorbent Assay (ELISA)

Colon and serum samples were diluted 1:10 in phosphate‐buffered saline (PBS, pH 7.4). ELISA was performed in triplicate using commercially available ELISA kits (Jianglai Biotechnology Co. Ltd., Shanghai, China) according to the manufacturer's protocols. After the stop solution was added to terminate the reaction, the absorbance of each well was measured at 450 nm using a microplate reader. The protein concentrations of the target analytes in the samples were calculated from a standard curve constructed using serial dilutions of a known concentration of the target protein.

### 2.13 Correlation Between Complement and Gut Microbiota Analysis

Complement levels in mouse colonic tissues were first measured by ELISA and used as the y‐axis. Subsequently, the relative abundance of gut microbiota in mouse intestinal contents was determined by 16S RNA sequencing and used as the x‐axis. Linear correlation analysis was conducted by integrating the two sets of experimental results described above.

### 2.14 Statistics Analysis

Data are expressed as means ± SEM and were analyzed using GraphPad Prism 9. For two‐group comparisons, an unpaired Student's *t*‐test or Mann–Whitney U test was applied. For multiple‐group assessments, a one‐way ANOVA was used for single‐factor analyses, and a two‐way ANOVA was used to evaluate interactions between two factors, with appropriate post hoc tests based on the experimental design. Statistical significance was classified as not significant (ns), with levels indicated as *p < 0.05, **p < 0.01, ***p < 0.001, and ****p < 0.0001.

## 3 Results

### 3.1 XYS1 Ameliorates Depression‐Like Behavior in CUMS Mice

**FIGURE 1:**
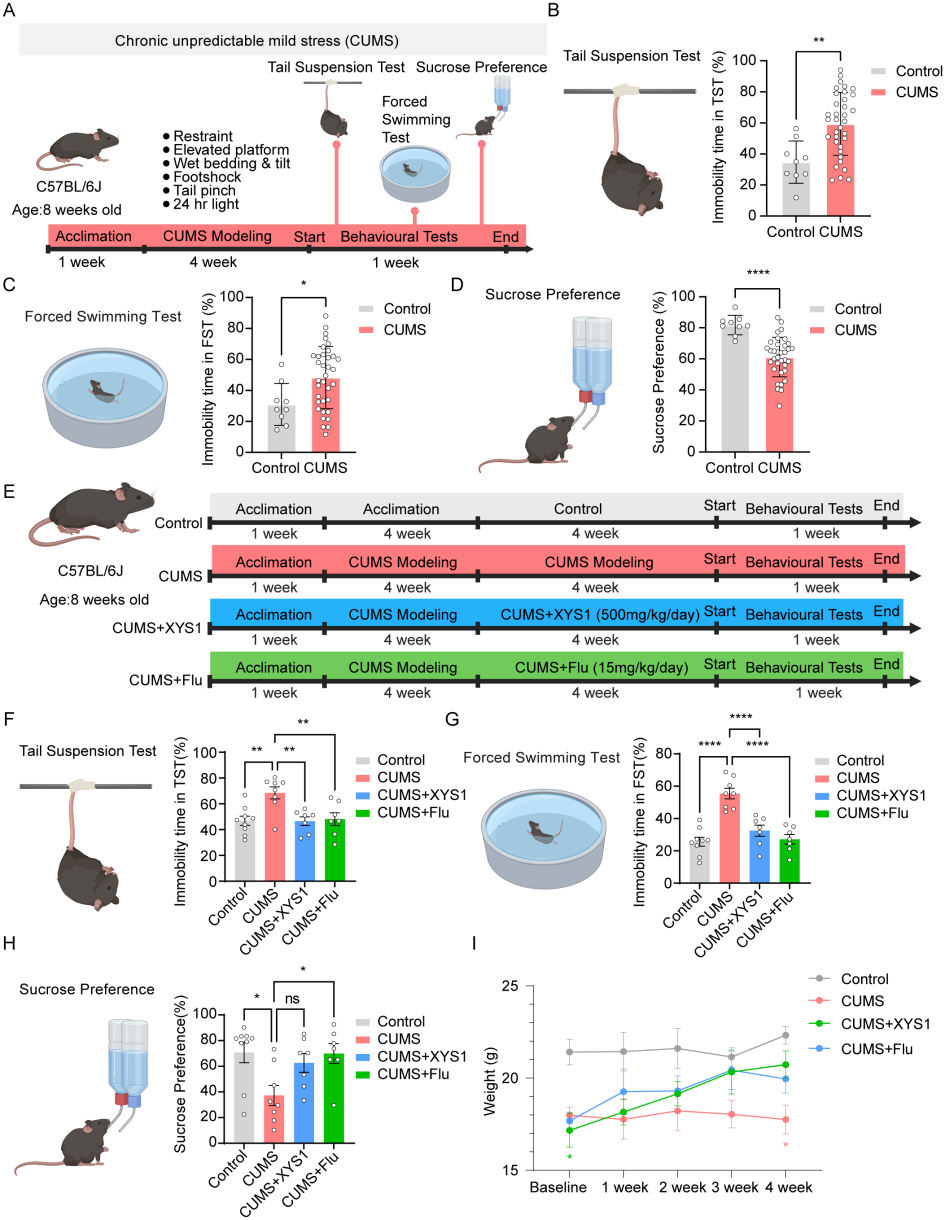
The American ginseng polysaccharide XYS1 can relieve CUMS‐induced depressive behavior. (A) Schematic timeline of chronic unpredictable mild stress (CUMS) mouse model and behavioral paradigms. Created with Biorender.com. (B, C) Immobility time in the tail suspension test (TST) (B) and the forced swimming test (FST) (C). (D) Quantification of sugar preference in the sucrose preference test (SPT). (E) Experimental timeline of administration of XYS1 and Flu in CUMS mice. Created with Biorender.com. (F, G) Immobility time in the tail suspension test (TST) (F) and the forced swimming test (FST) (G). (H) Quantification of sugar preference in the sucrose preference test (SPT). (I) Quantification of weight alteration. Data are represented as mea*n* ± SEM. Unpaired *t*‐test for (B, C) (*n* ≥ 9 per group). One‐way ANOVA with Dunnett's multiple comparisons test for (F–H) (*n* ≥ 7 per group), compared to CUMS group. Two‐way ANOVA with Tukey's multiple comparisons test for I (*n* ≥ 7 per group). Statistical significance as follows: ns (not significant), *p < 0.05, **p < 0.01, and ****p < 0.0001.

In our recent investigation, we have extracted and purified a polysaccharide, named XYS1, from *Panax quinquefolius Radix* via the enzymatic extraction and DEAE fractionation. To assess the antidepressant potential of XYS1 against chronic unpredictable mild stress (CUMS)‐induced depression‐like behaviors, we first established a CUMS model in mice, subjecting them to stressors over a four‐week period (Figure 1A). Upon confirmation of model establishment, we conducted a series of behavioral assays, including the tail suspension test (TST), forced swim test (FST), and sucrose preference test (SPT). In the TST, mice in the CUMS group exhibited a statistically significant increase in immobility duration (Figure 1B). Correspondingly, the FST results mirrored this trend, with the CUMS cohort showing prolonged immobility times (Figure 1C). Furthermore, the SPT revealed a significant reduction in the sucrose preference index among the CUMS group, substantiating the successful induction of depression‐like behavioral phenotypes (Figure 1D). Subsequently, we administered XYS1 to the depressed mice via gavage (Figure 1E). The dose of XYS1 (500 mg/kg/day) was determined based on the molecular weight equivalence to the positive control fluoxetine: Since fluoxetine (molecular weight 309.33 Da) was used at 15 mg/kg/day, the equivalent dose for XYS1 (indicated molecular weight 10,000 Da) was calculated to be approximately 500 mg/kg/day, so we initially only tested this single dose. Notably, treatment with XYS1 led to a significant amelioration of depression‐like behaviors compared to the CUMS group. This was evidenced by a marked reduction in immobility time in both the TST and FST in the CUMS+XYS1 group compared with the CUMS group (Figure 1F,G), alongside an elevated sucrose preference index in the SPT and a notable attenuation of body weight loss (Figure 1H,I). To further evaluate the multiple‐dose efficacy of XYS1, we added a low‐dose group (XYS1‐L, 50 mg/kg/day) for direct comparison with the high‐dose group (XYS1‐H, 500 mg/kg/day). The results showed that the low dose did not significantly alter CUMS‐induced increases in immobility time in the TST and FST, or decreases in sucrose preference (Figure S1). Collectively, these findings underscore the antidepressant properties of XYS1 and warrant further investigation into its pharmacological potential as a therapeutic agent.

### 3.2 XYS1 Reduces the Complement Activation in CUMS Mice and Cell Culture

**FIGURE 2:**
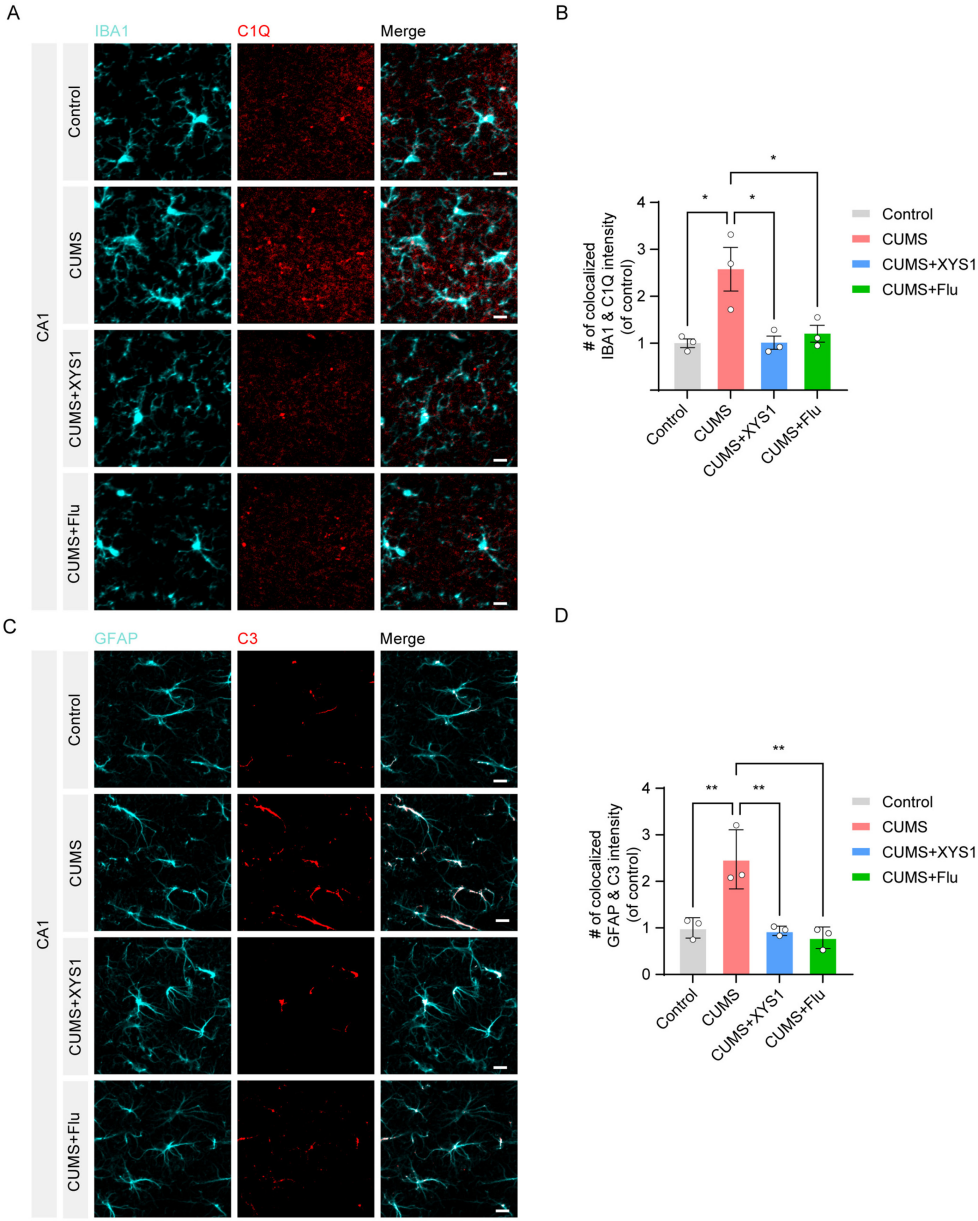
XYS1 alleviated CUMS‐induced increase of complement C1Q and C3 in CA1 hippocampus. (A, B) Immunofluorescence staining and quantification of IBA1‐labeled microglia (cyan) and C1Q (red) co‐localized in CA1 of the hippocampus. Scale bar = 10 μm. (C, D) Immunofluorescence staining of GFAP‐labeled astrocyte (cyan) and C3 (red) co‐stained in the CA1 area of the hippocampus. Scale bar = 10 μm. Data are represented as mean ± SEM. One‐way ANOVA with Tukey's multiple comparisons test for (B and D) (*n* = 3 per group). Statistical significance as follows: ns (not significant), *p < 0.05, and **p < 0.01.

To investigate the effect of XYS1 on the complement system in CUMS‐induced mice, we initially focused on the hippocampus, one of the critical brain regions involved in the regulation of emotion and mood‐related disorders such as depression. We examined the expression of complement proteins C1Q and C3 in the hippocampus using immunofluorescence staining (Figure 2). Our findings demonstrated a significant upregulation of complement C1Q specifically in microglia located in the CA1 subregion of the hippocampus (Figure 2A,B). In contrast, complement C3 expression was notably elevated in astrocytes within the same region (Figure 2C,D). Treatment with XYS1 or the positive control Fluoxetine effectively reduced the elevated levels of both C1Q and C3 (Figure 2). To further clarify the role of the complement system in depression, we additionally analyzed two other key emotion‐related brain regions, the medial prefrontal cortex (mPFC) and paraventricular nucleus of the hypothalamus (PVN) [25]. Consistent with the hippocampal results, our data showed that CUMS exposure significantly increased complement C1Q and C3 levels in both the mPFC and PVN, whereas XYS1 treatment markedly reversed these CUMS‐induced elevations (Figures S2 and S3). These results further confirm the widespread involvement of the C1Q/C3 signaling pathway in the pathological process of depression and the consistent regulatory effect of XYS1 on this pathway across multiple emotion‐related brain regions.

**FIGURE 3:**
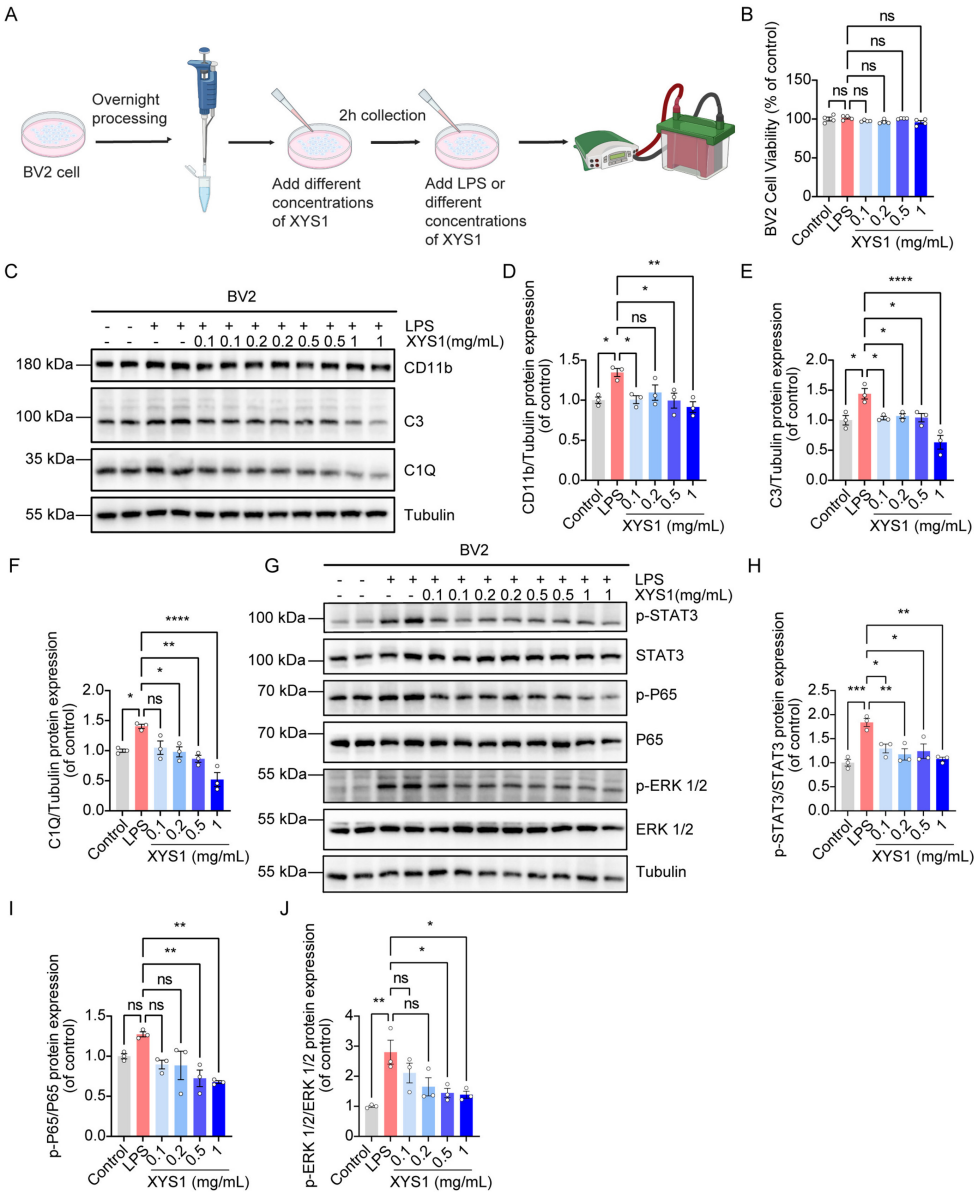
XYS1 reversed LPS‐induced activation of the C1Q/C3 complement pathway in BV2 cells. (A) Schematic representation of the treatment protocol utilizing varying concentration gradients of XYS1 on LPS‐stimulated BV2 microglial cells. Created with Biorender.com. (B) Quantification of cytotoxicity of XYS1 on BV2 cells. (C) Western blotting results for the expression levels of CD11b, C3, and C1Q proteins in BV2 microglial cells. (D–F) Quantification of the relative protein abundance of CD11b, C3, and C1Q compared to the control group. (G) Western blotting results for the expression levels of p‐STAT3, p‐P65, and p‐ERK 1/2 proteins in BV2 microglial cells. (H–J) Quantification of the relative protein abundance of p‐STAT3, p‐P65, and p‐ERK 1/2 compared to the control group. Data are represented as mean ± SEM. One‐way ANOVA with Tukey's multiple comparisons test for (B, D–F, and H–J) (*n* = 3 per group). Statistical significance as follows: ns (not significant), *p < 0.05, **p < 0.01, ***p < 0.001, and ****p < 0.0001.

To better understand how XYS1 alleviates depression, we conducted in vitro assays focused on the C1Q/C3 pathway. LPS was used to activate BV2 microglial cells, and various concentrations of XYS1 were applied (Figure 3A). The CCK‐8 assay confirmed no cytotoxicity (Figure 3B). Western blotting revealed that LPS significantly upregulated the expression of the microglial marker CD11b, along with complement components C1Q and C3. Treatment with XYS1 significantly reversed these changes (Figure 3C–F). Additionally, there was a notable increase in phosphorylated forms of key signaling molecules associated with the C1Q/C3 pathway, namely p‐STAT3, p‐P65, and p‐ERK1/2. However, treatment with XYS1 effectively mitigated these LPS‐induced alterations, restoring homeostasis in these signaling pathways, highlighting its potential therapeutic effects (Figure 3G–J).

### 3.3 XYS1 Attenuates Microglial Synapse Phagocytosis and Synapse Loss in CUMS Mice

**FIGURE 4:**
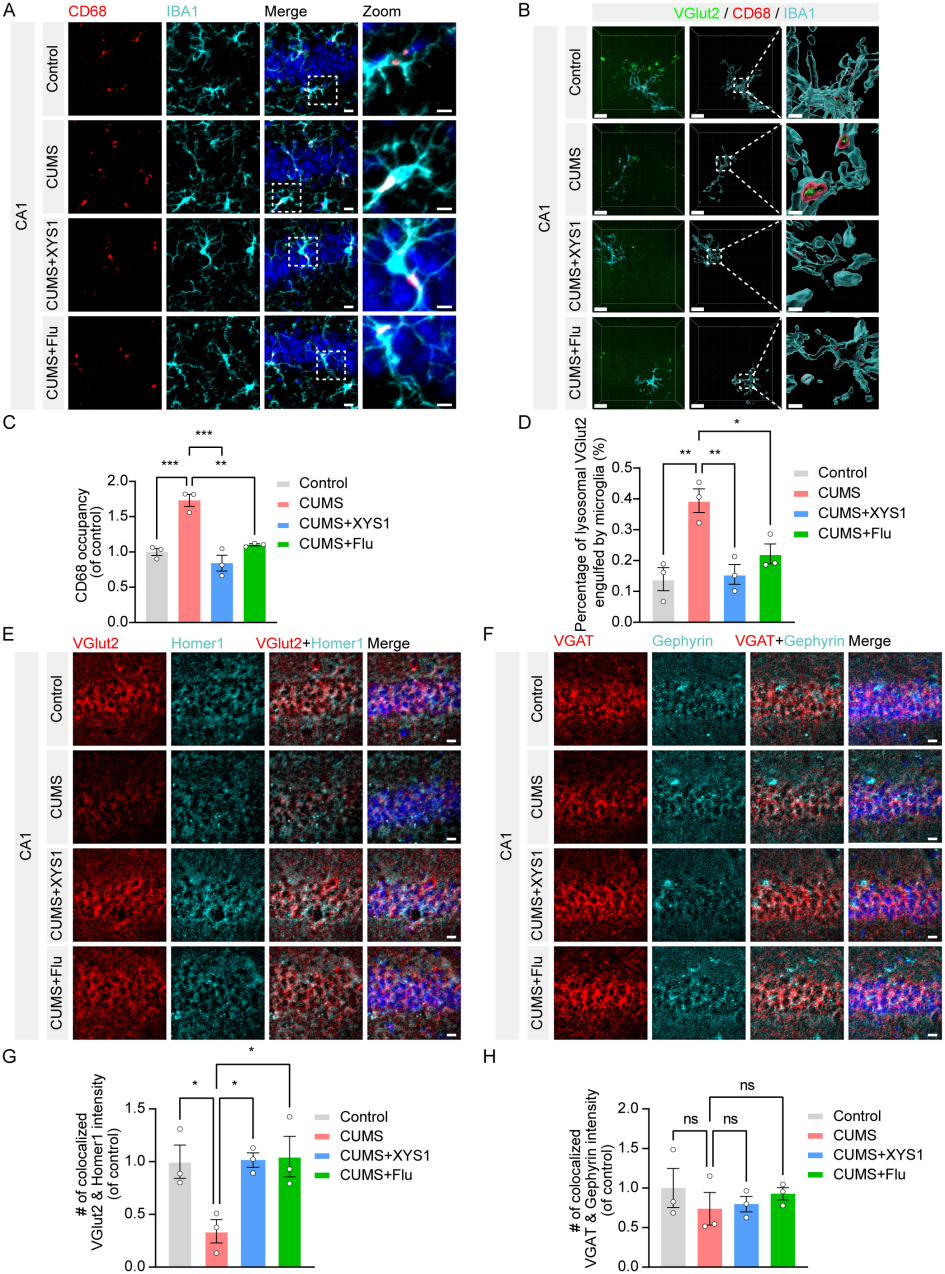
XYS1 alleviated CUMS‐induced microglia synaptic phagocytosis and excitatory synapse reduction. (A) Immunofluorescence staining of CD68 (red) in IBA1‐positive microglia (cyan) within the CA1 hippocampus, with nuclei labeled by DAPI (blue). Scale bar = 10 μm. Magnified image, Scale bar = 5 μm. (B) Schematic diagram of microglial phagocytosis of VGlut2 synapses in the CA1 region of the hippocampus. Left: Representative image showing co‐localization of IBA1‐positive microglia (cyan) with CD68‐positive lysosomes (red) and VGlut2 (green). Scale bar = 15 μm. Right: Corresponding magnified image. Scale bar = 3 μm. (C) Quantification of CD68 within IBA1 for (A). (D) Quantification of phagocytosis of VGlut2 synapse by microglia in CA1 based on (B). (E) Immunofluorescence staining of VGlut2/Homer1 synaptic density in the CA1 hippocampal region. Representative images showing co‐localized VGlut2 (red) and Homer1 (cyan), with nuclei labeled by DAPI (blue). Scale bar = 10 μm. (F) Immunofluorescence staining of VGAT/Gephyrin synaptic density in the CA1 hippocampal region. Representative images showing co‐localized VGAT (red) and Gephyrin (cyan), with nuclei labeled by DAPI (blue). Scale bar = 10 μm. (G) Quantification of colocalization of VGlut2 and Homer1 in the CA1 hippocampal region for (E). (H) Quantification of colocalization of VGAT and Gephyrin in the CA1 hippocampal region for (F). Data are represented as mean ± SEM. One‐way ANOVA with Tukey's multiple comparisons test for (C, D, G, and H) (*n* = 3 per group). Statistical significance as follows: ns (not significant), *p < 0.05, **p < 0.01, and ***p < 0.001.

The complement system plays a crucial role in modulating synaptic pruning by microglia within the brain [25]. To evaluate microglial activation, we performed immunohistochemical analyses using IBA1 and CD68 on brain sections (Figure 4A,C). Our findings show that induction of CUMS significantly increased CD68 expression in microglia located in the hippocampal CA1 region of mice. This increase can be substantially attenuated through treatment with XYS1 (Figure 4C). To assess synaptic phagocytosis by microglia in the hippocampal CA1 region of mice subjected to chronic stress, we stained brain sections with IBA1, CD68, and the presynaptic marker VGlut2 (Figure 4B,D). We quantified the extent of synaptic phagocytosis by measuring the co‐localization of the three markers (IBA1/CD68/VGlut2), revealing that CUMS markedly enhanced microglial‐mediated synaptic phagocytosis. Notably, treatment with XYS1 mitigated this aberrant microglial synaptic pruning (Figure 4D). To investigate the relationship between improved phagocytosis and alterations in synaptic density in the hippocampus under chronic stress conditions, we conducted immunofluorescence staining of brain sections targeting excitatory synapses and inhibitory synapses. Our findings from the CUMS model demonstrated a significant reduction in VGlut2/Homer1 synaptic density within the CA1 region of the hippocampus (Figure 4E,G). In contrast, the density of VGAT/Gephyrin synapses remained unchanged (Figure 4F,H). Notably, treatment with XYS1 effectively reversed the decline in excitatory synapse density within the hippocampal CA1 region (Figure 4E–H). We further detected the effect of XYS1 on CUMS‐induced changes in synaptic density in the medial prefrontal cortex (mPFC). Consistent with hippocampal results, CUMS exposure significantly reduced VGlut2/Homer1 synaptic density in the mPFC, whereas treatment with either XYS1 or the positive control Fluoxetine markedly ameliorated this CUMS‐induced reduction (Figure S4). Moreover, we tested the impact of CUMS and XYS1 treatment on the density of glutamatergic (Glu) and GABAergic (GABA) neurons in the hippocampus using immunofluorescence staining (Figure S5). CUMS exposure led to a significant reduction in the fluorescence intensity of Glu‐positive neurons compared with the control group (Figure S5A,B), indicating a loss of glutamatergic neurons. Treatment with either XYS1 or Flu effectively reversed this CUMS‐induced reduction, restoring Glu neuron density to levels comparable to the control group. In contrast, the fluorescence intensity of GABA‐positive neurons remained unchanged across all experimental groups (Figure S5C,D), suggesting that XYS1 treatment selectively affects glutamatergic, but not GABAergic, neuronal density in the hippocampus. Collectively, these results indicate that XYS1 exerts a protective effect by mitigating microglial dysfunction and preserving glutamatergic neurons and excitatory synapses, which may contribute to the restoration of synaptic plasticity and the alleviation of depressive‐like behaviors in CUMS mice.

### 3.4 XYS1 Restores the Homeostasis of Gut Microbiota Perturbed by CUMS

**FIGURE 5:**
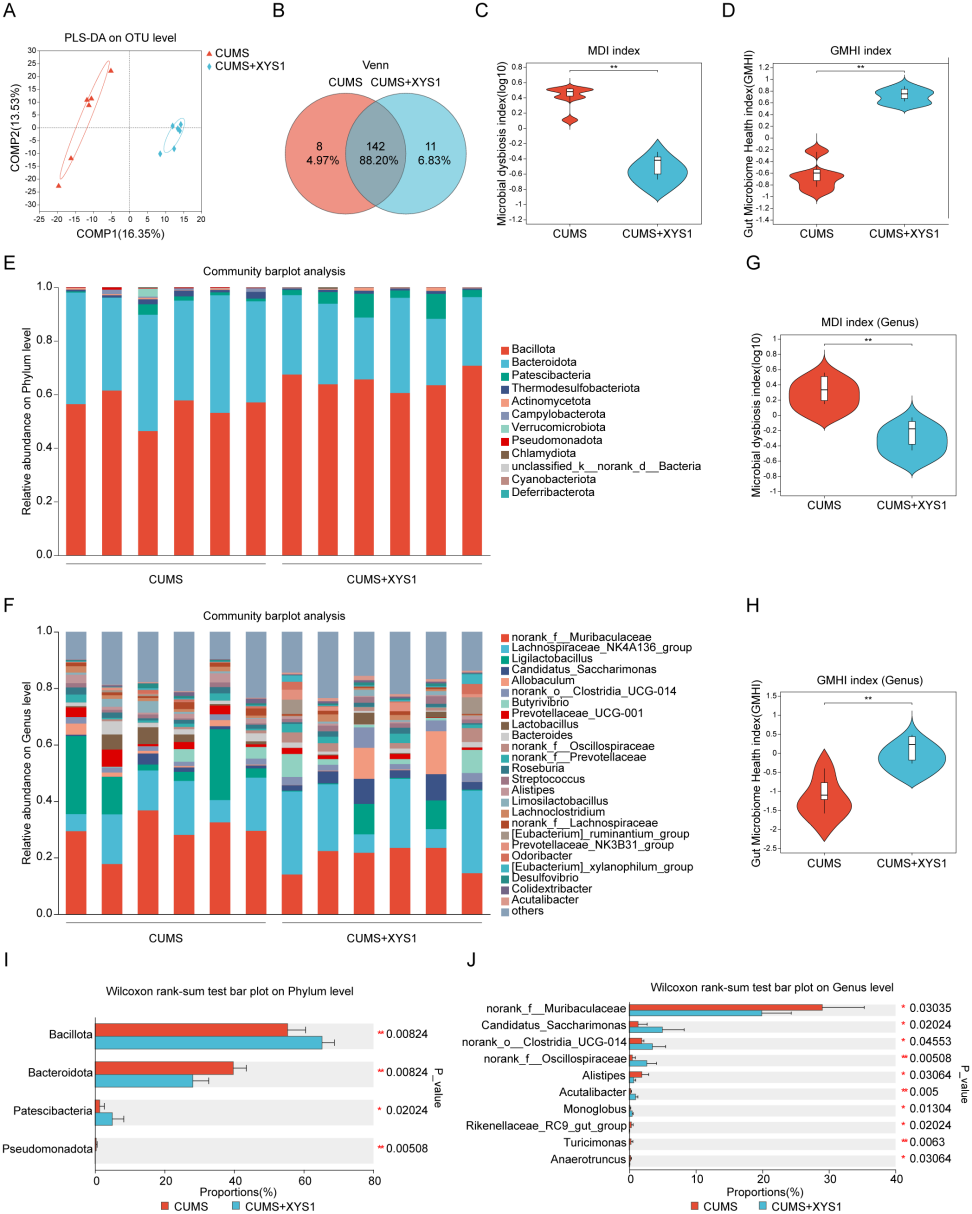
XYS1 modified gut microbiota and mitigated CUMS‐induced dysbiosis. (A) Beta diversity was evaluated by partial least squares discriminant analysis (PLS‐DA). (B) A Venn diagram of the OTU level. (C) Microbiota Dysbiosis Index (MDI) at the OTU level. (D) Gut Microbiota Health Index (GMHI) at the OTU level. (E, F) Distribution of intestinal microbiota in mouse intestinal contents at the phylum level (E) and genus level (F). (G) Microbiota Dysbiosis Index (MDI) at the genus level. (H) Gut Microbiota Health Index (GMHI) at the genus level. (I) Quantification of microbial flora at the phylum level. (J) Quantification of microbial flora at the genus level. Data are represented as mean ± SEM. Mann–Whitney U test for (C, D, G, H, I, and J) (*n* = 6 per group). Statistical significance as follows: ns (not significant), *p < 0.05, and **p < 0.01.

To examine the impact of XYS1 on gut microbiota in mice subjected to CUMS, we performed 16S rRNA sequencing on the intestinal contents. Partial Least Squares‐Discriminant Analysis (PLS‐DA) indicated a significant separation between the CUMS+XYS1 cohort and the CUMS‐only group at the operational taxonomic unit (OTU) level (Figure 5A). Venn diagram analysis revealed that 142 OTUs were shared between the two groups (88.2%), alongside 8 unique OTUs in the CUMS group (4.97%) and 11 unique OTUs in the CUMS+XYS1 group (6.83%), (Figure 5B). Further assessment using the Microbiota Dysbiosis Index (MDI) and Gut Microbiota Health Index (GMHI) at the OTU level showed that XYS1 administration significantly decreased MDI values and enhanced GMHI scores relative to the CUMS group (Figure 5C,D). A detailed species composition analysis identified 12 phyla, with *Bacillota*, *Bacteroidota*, and *Patescibacteria* constituting the predominant components of the mouse fecal microbiota (Figure 5E,F). At the genus level, administration of XYS1 polysaccharide from American ginseng significantly reduced MDI values in CUMS mice while markedly increasing GMHI levels (Figure 5G,H). The XYS1 treatment notably increased the abundance of *Bacillota* and *Patescibacteria* while decreasing Bacteroidota levels in comparison to the CUMS group (Figure 5I). At the genus level, XYS1 administration led to a significant reduction in the relative abundance of *norank_f__Muribaculaceae and Alistipes*, while significantly enhancing the presence of *Candidatus_Saccharimonas*, *norank_o__Clostridia_UCG‐014*, and *norank_f__Oscillospiraceae* (Figure 5J). These results underscore XYS1's ability to restore gut microbiota homeostasis disrupted by CUMS.

### 3.5 XYS1 Attenuates CUMS‐Induced Increases in Intestinal Complement C1Q and C3

**FIGURE 6:**
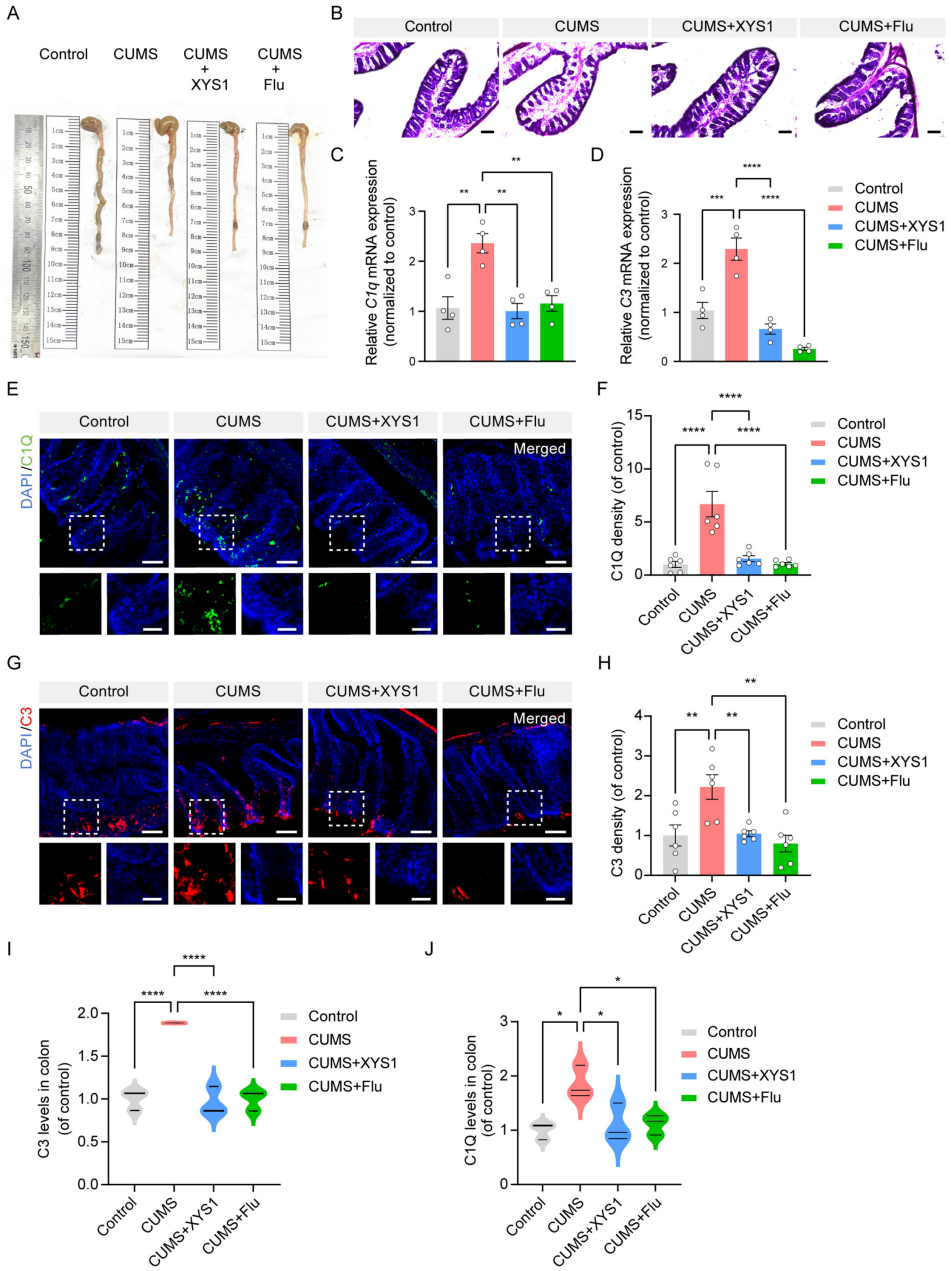
XYS1 reduced CUMS‐induced colonic complement overexpression. (A) Schematic diagram of colon and cecum length measurements. (B) H&E staining of colon tissues. Scale bar = 25 μm. (C, D) Quantitative analysis of *C1q* (C) and *C3* (D) mRNA expression in the colon. (E, F) Immunofluorescence staining and quantification of C1Q (green) expression in colon, with nuclei labeled by DAPI (blue). Scale bar = 50 μm. Magnified view Scale bar = 12.5 μm. (G, H) Immunofluorescence staining and quantification of C3 (red) expression in colon, with nuclei labeled by DAPI (blue). Scale bar = 50 μm. Magnified view Scale bar = 12.5 μm. (I, J) Enzyme‐linked immunosorbent assay and quantification of C3 (I) and C1Q (J) expression in colon. Data are represented as mean ± SEM. One‐way ANOVA with Tukey's multiple comparisons test for (C, D, I, and J, *n* = 4 per group; F and H, *n* = 6 per group). Statistical significance as follows: ns (not significant), *p < 0.05, **p < 0.01, ***p < 0.001, and ****p < 0.0001.

To investigate colonic pathology related to depression, we examined colon tissue from mice. Control mice displayed intact colon structures with closely arranged glands. In contrast, colon tissues from CUMS mice showed broken epithelial cells, destroyed mucosal layers, reduced gland numbers, loss of cup cells, and irregular crypt surfaces. However, after XYS1 treatment, colon structure improved significantly, with increased cup cells and normalized gland arrangement (Figure 6A,B). A recent study investigated the local synthesis of complement proteins within the gastrointestinal tract [34]. To assess the potential impact of XYS1 on intestinal complement activation, we systematically evaluated the mRNA and protein levels of C1Q and C3 in colonic tissues. First, qRT‐PCR analysis revealed that CUMS exposure significantly upregulated C1Q and C3 gene expression in the colon compared with controls (Figure 6C,D). Immunofluorescence staining confirmed elevated protein levels of C1Q and C3 in the colonic mucosa of CUMS‐induced mice (Figure 6E–H), with distinct localization in intestinal epithelial and stromal cells, as previously reported [34]. To further validate these findings, we performed an ELISA on colonic tissue homogenates, which quantitatively demonstrated that CUMS mice exhibited markedly increased protein concentrations of C1Q and C3 relative to controls. Importantly, treatment with *Panax quinquefolius* polysaccharide XYS1 consistently reversed the CUMS‐induced upregulation of C1Q and C3 at both mRNA and protein levels (Figure 6C–J). These convergent results strongly confirm that XYS1 effectively suppresses CUMS‐induced aberrant complement activation in the colon.

To further clarify the association between XYS1‐mediated gut microbiota regulation and alterations in intestinal complement C1Q and C3, we performed linear correlation analyses between key gut microbial phyla, Bacillota, Bacteroidota, and colonic complement C1Q and C3 levels (Figure S6). In the CUMS and CUMS‐XYS1 groups, colonic C1Q and C3 levels showed a strong negative correlation with Bacillota abundance and a positive correlation with Bacteroidota abundance. These results demonstrate that XYS1‐mediated microbiota remodeling, characterized by enriched Bacillota and reduced Bacteroidota, is significantly correlated with the attenuation of CUMS‐induced complement C1Q and C3 overactivation in the colon. These findings suggest a potential link between gut microbiota reshaping and intestinal complement regulation by XYS1.

### 3.6 XYS1 Mitigates CUMS‐Induced Colonic and Systemic Inflammation

**FIGURE 7:**
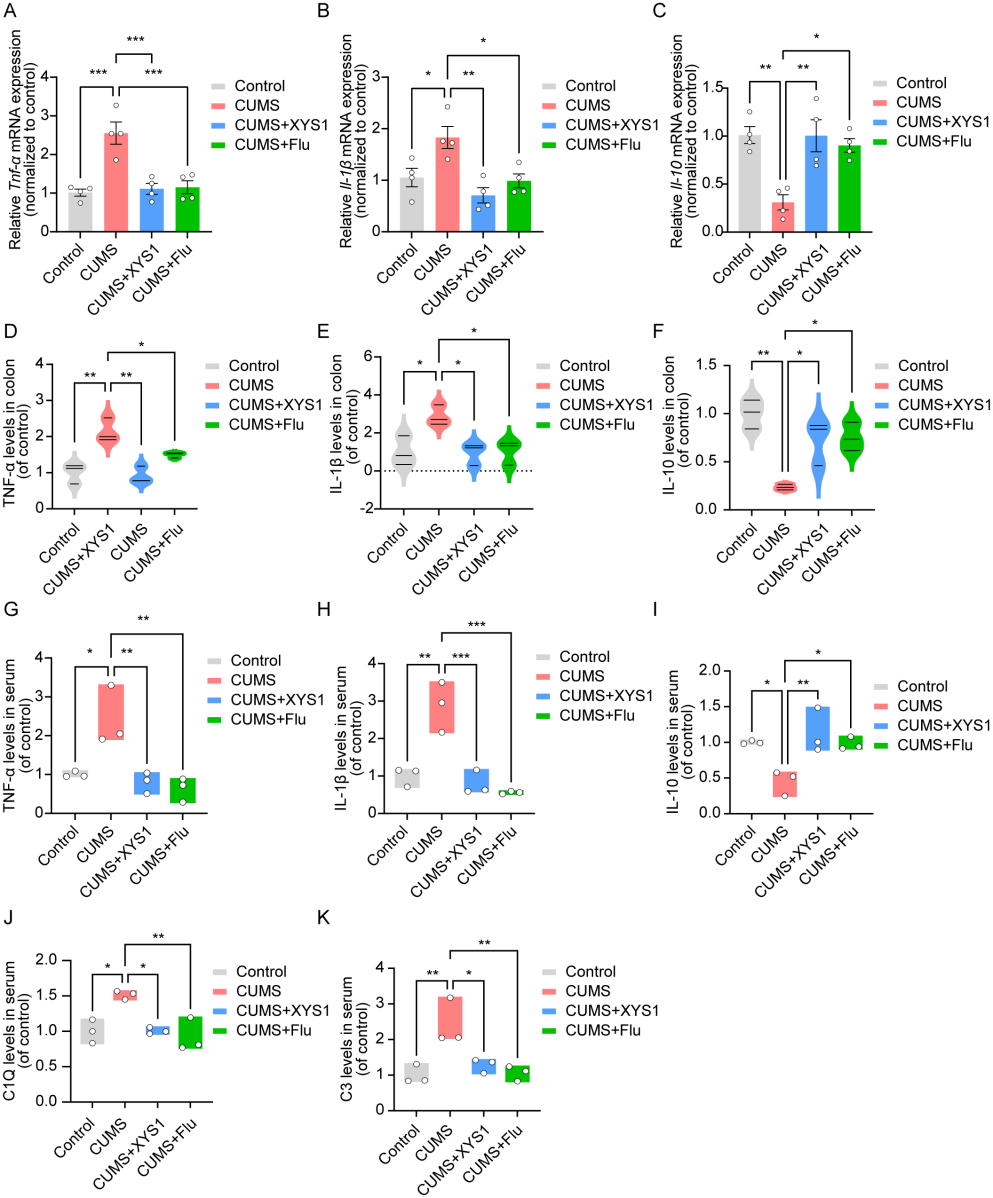
XYS1 reduced CUMS‐induced inflammatory responses. (A–C) Quantitative analysis of *Tnf‐α* (A), Il‐1β (B), and *Il‐10* (C) mRNA expression in the colon. (D–F) Enzyme‐linked immunosorbent assay and quantification of TNF‐α, IL‐1β, and IL‐10 expression in colon. (G–K) Enzyme‐linked immunosorbent assay and quantification of TNF‐α, IL‐1β, IL‐10, C1Q, and C3 expression in serum. Data are represented as mean ± SEM. One‐way ANOVA with Tukey's multiple comparisons test for (A–C, *n* = 4 per group; D–K, *n* = 3 per group). Statistical significance as follows: ns (not significant), *p < 0.05, **p < 0.01, and ***p < 0.001.

The gut‐brain axis (GBA) is a bidirectional communication network integrating neural, endocrine, and immune signaling, with the gut microbiota as a pivotal mediator. Within this axis, complement activation and pro‐inflammatory/anti‐inflammatory responses form a reciprocal regulatory loop that dynamically modulates GBA homeostasis. To comprehensively assess CUMS‐induced alterations in colonic pathology and systemic inflammation, we evaluated inflammatory cytokine levels in colonic tissues and serum, as well as serum complement components. First, qRT‐PCR results showed that CUMS exposure significantly upregulated the mRNA levels of the pro‐inflammatory cytokines *Tnf‐α* and *Il‐1β* in colonic tissues, while concurrently inhibiting *Il‐10* expression (Figure 7A–C). Consistent with these transcriptional changes, ELISA quantification revealed that CUMS mice exhibited markedly increased protein levels of TNF‐α and IL‐1β, as well as reduced IL‐10 protein levels in the colon, all of which were effectively reversed by XYS1 administration (Figure 7D–F).

Given that intestinal inflammatory and complement alterations can propagate systemically to affect the central nervous system, we further measured serum levels of proinflammatory and anti‐inflammatory cytokines and complement components by ELISA. Consistent with the colonic inflammatory profile, CUMS mice showed elevated serum levels of pro‐inflammatory cytokines TNF‐α, IL‐1β, and complement proteins C1Q and C3, accompanied by a significant decrease in the anti‐inflammatory cytokine IL‐10 (Figure 7G–K). These systemic changes were also mitigated by XYS1 treatment, reinforcing its role in modulating the gut‐brain axis via regulating peripheral inflammation and complement activation.

## 4 Discussion

Depression, classified as a mood disorder, manifests through anhedonia and pervasive sadness and constitutes a significant public health concern globally. Studies indicate a link between synaptic changes and depression, with reductions in synaptic density, dendritic spines, boutons, and glial cells noted in stressed individuals [35, 36, 37]. Notably, some drugs, like ketamine, may promote rapid synaptic restoration. Thus, investigating the mechanisms driving this synaptic loss may pave the way for innovative therapeutic approaches to ameliorate depressive symptoms. Recent investigations have underscored the pivotal role of the complement system in mediating synapse loss through microglial synaptic engulfment. The interaction between the complement system and microglia is a critical pathway implicated in synaptic degradation across a range of neuropathological conditions. In a murine model of LPS‐induced depression, synaptic loss and dysregulated microglial phagocytosis were observed in the hippocampal dentate gyrus (DG). The complement pathways, specifically C1Q and C3‐CR3 signaling, have been implicated in mediating the aberrant phagocytic activity of microglia toward synapses in this LPS‐ and stress‐driven depressive state [27, 33]. In our study, the results demonstrate that chronic stress activates the C1Q/C3 complement pathway in the hippocampal CA1 region, with distinct increases in C1Q in microglia and C3 in astrocytes. XYS1 effectively ameliorates depression‐like pathology by suppressing this aberrant complement activation. The in vitro findings further elucidate that XYS1 exerts its therapeutic effect, at least in part, by inhibiting microglial activation and downregulating the C1Q/C3 pathway via modulation of STAT3, NF‐κB (P65), and ERK1/2 signaling. Therefore, targeting the C1Q/C3 complement cascade is a key mechanism by which XYS1 mitigates aberrant microglial‐mediated synaptic pruning, ultimately preventing synaptic loss.

Recent investigations have intensified focus on the bidirectional interaction between gastrointestinal microbiota and the central nervous system. A recent study showed that microbiota manipulation affects microglia‐mediated synaptic pruning and dendritic spine remodeling, leading to behavioral anomalies [38]. Ginseng shows promising potential to mitigate dysbiosis‐associated disorders, particularly in the gastrointestinal tract and central nervous system, by re‐establishing microbial equilibrium and enhancing gut barrier integrity. Its therapeutic properties include promoting beneficial probiotic populations such as *Firmicutes*, *Lactobacillus*, *Bifidobacterium*, and *Akkermansia*, while concurrently reducing the load of pathogenic bacteria like *Helicobacter*, *Clostridium*, and *Proteobacteria* [39]. Previous research indicates that orally administered ginseng interacts with the gut microbiota, leading to the metabolism of its hydrophilic components, particularly ginsenosides, into hydrophobic metabolites through gastric juice and microbial action. Notably, these metabolites demonstrate enhanced pharmacological activities, exhibiting antitumor, anti‐inflammatory, antidiabetic, antiallergic, and neuroprotective effects when compared to their parent ginsenosides [40, 41]. Research directly linking ginseng's effects on microbial communities to gut health remains limited, particularly in relation to neuropsychiatric disorders. In our investigation, administration of *Panax quinquefolius* polysaccharide XYS1 induced substantial remodeling of the gut microbial community in CUMS‐exposed mice, which was closely associated with marked amelioration of colonic pathology. This includes the restoration of epithelial integrity, normalization of glandular architecture, and attenuation of mucosal damage. Compositionally, XYS1 increased the abundances of *Bacillota* and *Patescibacteria* while reducing *Bacteroidota*. At the genus level, it significantly decreased pro‐inflammatory taxa such as *norank_f__Muribulaceae* and *Alistipes*, and promoted beneficial genera including *Candidatus_Saccharimonas*, *norank_o__Clostridia_UCG‐014*, and *norank_f__Oscillospiraceae*. Collectively, these results demonstrate that XYS1 not only restores gut microbiota homeostasis disrupted by CUMS but also correlates with structural and functional improvements in intestinal health, providing a mechanistic basis for its potential application in microbiota‐targeted therapies for stress‐related disorders.

The complement system, a core component of innate immunity, is widely expressed in intestinal epithelial cells, stromal cells, immune cells, and the CNS microglia, astrocytes, and neurons. In the gut, commensal microbiota dynamically modulate complement expression [28]. Physiological levels of complement maintain intestinal barrier integrity by clearing apoptotic cells and pathogens [34], while gut dysbiosis increases pro‐inflammatory responses, triggering aberrant complement activation. Notably, complement status may also modulate gut microbiota composition, a relationship that was not explored in early investigations using C3‐ and CD11b‐deficient transgenic mice to dissect the association between complement signaling and synaptic pruning. Specifically, adult mice lacking C3 have been shown to have reduced abundance of Bacteroidetes and increased abundance of Firmicutes [42, 43], two dominant gut microbial phyla known to be key producers of specific short‐chain fatty acids (SCFAs). These SCFAs are critically involved in regulating microglial maturation and function, as well as maintaining blood–brain barrier (BBB) integrity [44], thus linking gut microbiota‐complement crosstalk to CNS homeostasis. Previous studies revealed that gut microbiota transplantation from CUMS‐induced depressed mice to specific pathogen‐free and germ‐free mice induced depression‐like behaviors in the recipients [45]. Enhanced activation of the complement C3/CR3 pathway and abnormal microglia‐mediated synaptic pruning in the prefrontal cortex were observed. However, antidepressants and fecal microbiota transplantation from treated donors improved depression‐like behaviors, corrected gut microbiome disturbances, inhibited the complement pathway, and increased synapsin and PSD95 levels [28]. Consistent with these findings, our study reveals that CUMS exposure induces dysbiosis of the gut microbiota and significantly upregulates mRNA and protein levels of C1Q and C3 in colonic tissues, as assessed by qRT‐PCR, immunofluorescence staining, and ELISA. Immunofluorescence staining further localized the elevated C1Q and C3 proteins to intestinal epithelial and stromal cells, consistent with previous reports that stromal cells are the main local producers of complement C3 in the gut [34]. This peripheral complement overexpression not only exacerbates intestinal mucosal inflammation but also contributes to systemic complement activation; peripheral complement components can cross the blood–brain barrier (BBB) under pathological conditions, thereby directly affecting CNS complement pathways. Consistent with this, our study found that CUMS‐induced upregulation of colonic C1Q and C3 was accompanied by elevated serum levels of these two complement proteins, indicating that aberrant intestinal complement activation can propagate systemically. Crucially, treatment with *Panax quinquefolius* polysaccharide XYS1 effectively reversed these CUMS‐induced pathological changes. Compared with the CUMS group, XYS1 effectively mitigated the CUMS‐induced upregulation of mRNA and protein levels of the complement proteins C1Q and C3 in colonic tissues and mitigated elevated serum complement levels, highlighting its regulatory role in peripheral complement activation. Meanwhile, XYS1 treatment notably increased the abundance of *Bacillota* but decreased Bacteroidota levels compared with the CUMS group. Collectively, these observations indicate bidirectional crosstalk between gut microbiota composition and colonic complement activation, dynamically modulated by XYS1. Specifically, the XYS1‐mediated enrichment of Bacillota, alongside reduced Bacteroidota abundance, correlates with the suppression of colonic C1Q and C3 upregulation in CUMS mice. It is plausible that the XYS1‐induced shift toward Bacillota dominance contributes to the downregulation of colonic C3. Conversely, the reduction in Bacteroidota, whose abundance has been linked to increased intestinal inflammation [46, 47, 48], may be associated with complement activation in pathological states of the colon. This aligns with prior evidence that commensal microbiota dynamically tune gut‐localized C3 expression [34], with compositional shifts directly impacting complement‐mediated mucosal immunity. Thus, our findings suggest that XYS1 modulates the gut microbiota‐complement axis by reshaping key bacterial phyla Bacillota and Bacteroidota, which are associated with aberrant colonic C1Q and C3 activation, a critical step linking peripheral immune homeostasis to central neuroprotective effects.

Complement activation and pro‐inflammatory and anti‐inflammatory responses form a reciprocal regulatory loop in the GBA. On the one hand, pro‐inflammatory cytokines such as TNF‐α, IL‐6, and IL‐1β induced by gut dysbiosis or mucosal damage upregulate intestinal complement expression [28], thereby amplifying local and systemic inflammation. In our study, CUMS exposure simultaneously increased the mRNA and protein levels of pro‐inflammatory cytokines TNF‐α and IL‐1β in the colon, inhibited the anti‐inflammatory cytokine IL‐10, and upregulated complement C1Q and C3 expression. XYS1 treatment reversed these changes in both inflammatory cytokines and complement components in a consistent manner. On the other hand, complement activation can promote pro‐inflammatory responses. Complement fragments bind to their receptors on immune cells and intestinal epithelial cells, triggering the release of pro‐inflammatory cytokines and chemokines, which further disrupt gut barrier function and exacerbate microbiota dysbiosis [49]. Conversely, anti‐inflammatory cytokines, such as IL‐10, suppress inflammation to restore mucosal homeostasis [50]. Our results show that XYS1 can upregulate IL‐10 levels in CUMS mice and inhibit pro‐inflammatory cytokines and complement activation, suggesting that XYS1 may break the vicious cycle between complement overactivation and pro‐inflammatory responses, thereby restoring intestinal mucosal immunity and systemic immune homeostasis. Furthermore, the consistency of inflammatory and complement changes in colonic tissues and serum observed in our study confirms that intestinal immune abnormalities can spread systemically, and XYS1 can modulate the gut‐brain axis by regulating this peripheral immune‐complement network. This provides direct experimental evidence for the interaction among gut complement activation, inflammation, and dysbiosis of the gut microbiota, and clarifies the potential mechanism by which XYS1 exerts its antidepressant effect by targeting the gut‐brain‐complement axis. Collectively, the above findings demonstrate that XYS1 exerts protective effects on intestinal mucosal immunity and systemic immune homeostasis, likely mediated through its influence on gut microbiota. This provides a significant link between gut dysbiosis, complement activation, intestinal inflammatory imbalance, and the pathophysiology of depressive disorders, highlighting a potential mechanism by which gut‐targeted interventions like XYS1 could impact depression.

## 5 Conclusions

In this study, our findings indicate that XYS1, a polysaccharide from *Panax quinquefolius Radix*, alleviates depression‐like behaviors in CUMS mice, as demonstrated by improved behavioral performance and reduced weight loss. Mechanistically, XYS1 inhibits complement pathway activation, lowering C1Q and C3 levels not only in the hippocampus but also in the mPFC and PVN regions, which reduces microglial hyperactivation and synaptic phagocytosis, thereby selectively preserving glutamatergic neuron and excitatory synapse density in both the hippocampus. Additionally, XYS1 modulates gut microbiota and mitigates CUMS‐induced dysbiosis, characterized by enriched Bacillota and reduced Bacteroidota, which is significantly correlated with attenuation of intestinal complement C1Q and C3 overactivation. suggesting its involvement in gut–brain axis regulation. Collectively, these findings highlight the multifaceted antidepressant effects of XYS1 and support its potential as a therapeutic agent targeting complement‐driven neuroimmune and gastrointestinal pathways in depression.

## Author Contributions

**Mengjun Xie**, **Lingling Feng:** data curation; formal analysis; investigation; methodology; visualization; writing – original draft preparation. **Ruyi Li:** investigation; methodology. **Mengqi Li**, **Liang Shen**, **Minghui Zhang**, **Yutong Wei**, **Qiao Yin**, **Datong Wang**, **Liqing Chen:** investigation; methodology. **Kai Song:** funding acquisition; supervision; writing – review and editing. **Peipei Wang:** conceptualization; funding acquisition; project administration; supervision; writing – review and editing. **Qifei Cong:** conceptualization; funding acquisition; project administration; supervision; writing – original draft preparation; writing – review and editing. All authors reviewed and approved the final manuscript.

## Funding

This work was supported by the National Natural Science Foundation of China (32471014, 32200778); the Key Project of the Natural Science Foundation of Jiangsu Provincial Higher Education Institutions (25KJA310003); the Natural Science Foundation of Jiangsu Province (BK20220494); the Suzhou Medical and Health Technology Innovation Project (SKY2022107); the State Key Laboratory of Drug Research, Chinese Academy of Sciences (SKLDR‐2023‐KF‐05); the Young Science Talents Promotion Project of Jiangsu Science and Technology Association (TJ‐2023‐019); the Suzhou International Joint Laboratory for Diagnosis and Treatment of Brain Diseases; the Priority Academic Program Development of Jiangsu Higher Education Institutes; the Project of MOE Key Laboratory of Geriatric Diseases and Immunology (KJS2501); the Jiangsu Key Laboratory of Drug Discovery and Translational Research for Brain Diseases (KJS2546); the Project of Biomedical Basic Research Center of Jiangsu and the Scientific Research Project of Jiangsu Association of Chinese Medicine (ZXF2024072).

## Conflicts of Interest

The authors declare no conflicts of interest.

## Supporting information


**Figure S1:** XYS1 relieved CUMS‐induced depressive behavior in a dose‐dependent manner.
(A) Experimental timeline of administration of XYS1‐L and XYS1‐H in CUMS mice. Created with Biorender.com. (B, C) Immobility time in the tail suspension test (TST) (B) and the forced swimming test (FST) (C). (D) Quantification of sugar preference in the sucrose preference test (SPT). Data are represented as mean ± SEM. One‐way ANOVA with Dunnett's multiple comparisons test for (B‐D) (*n* ≥ 7 per group, data of XYS1‐H is related to Figure 1F–H), compared to CUMS group. Statistical significance as follows: ns (not significant), **p < 0.01, ***p < 0.001, ****p < 0.0001.

**Figure S2:** XYS1 alleviated CUMS‐induced increase of complement C1Q and C3 in the mPFC.
(A, B) Immunofluorescence staining and quantification of IBA1‐labeled microglia (cyan) and C1Q (red) co‐localized in the mPFC. Scale bar = 10 μm. (C, D) Immunofluorescence staining of GFAP‐labeled astrocyte (cyan) and C3 (red) co‐stained in the mPFC. Scale bar = 10 μm. Data are represented as mean ± SEM. One‐way ANOVA with Tukey's multiple comparisons test for (B and D) (*n* = 3 per group). Statistical significance as follows: ns (not significant), *p < 0.05, **p < 0.01.

**Figure S3:** XYS1 alleviated CUMS‐induced increase of complement C1Q and C3 in the PVN.
(A, B) Immunofluorescence staining and quantification of IBA1‐labeled microglia (cyan) and C1Q (red) co‐localized in the PVN. Scale bar = 10 μm. (C, D) Immunofluorescence staining of GFAP‐labeled astrocyte (cyan) and C3 (red) co‐stained in the PVN. Scale bar = 10 μm. Data are represented as mean ± SEM. One‐way ANOVA with Tukey's multiple comparisons test for (B and D) (*n* = 3 per group). Statistical significance as follows: ns (not significant), *p < 0.05, **p < 0.01, ***p < 0.001.

**Figure S4:** XYS1 ameliorated CUMS‐induced excitatory synapse reduction in the mPFC.
(A) Immunofluorescence staining of VGlut2/Homer1 synaptic density in the mPFC. Representative images showing co‐localized VGlut2 (red) and Homer1 (cyan), with nuclei labeled by DAPI (blue). Scale bar = 10 μm. (B) Quantification of colocalization of VGlut2 and Homer1 in the mPFC region for (A). Data are represented as mean ± SEM. One‐way ANOVA with Tukey's multiple comparisons test for (B) (*n* = 3 per group). Statistical significance as follows: ns (not significant), *p < 0.05, **p < 0.01.

**Figure S5:** XYS1 attenuated CUMS‐induced reduction in glutamatergic neurons in the hippocampus.
(A, B) Immunofluorescence staining and quantification of glutamatergic neurons (Glu, green) in the hippocampus. Scale bar = 10 μm. (C, D) Immunofluorescence staining of GABAergic neurons (GABA, green) in the hippocampus. Scale bar = 10 μm. Data are represented as mean ± SEM. One‐way ANOVA with Tukey's multiple comparisons test for (B and D) (*n* = 3 per group). Statistical significance as follows: ns (not significant), ***p < 0.001, ****p < 0.0001.

**Figure S6:** Correlation analysis of key gut microbial phyla with colonic complement C1Q and C3 levels in CUMS mice after XYS1 treatment.
(A) Linear correlation analysis of complement C1Q with Bacillota in mouse gut microbiota; R^2^ (CUMS) = 0.7757, R^2^ (CUMS+XYS1) = 0.9244. (B) Linear correlation analysis of complement C3 with Bacillota in mouse gut microbiota; R^2^ (CUMS) = 0.8334, R^2^ (CUMS+XYS1) = 0.9188. (C) Linear correlation analysis of complement C1Q with Bacteroidota in mouse gut microbiota; R^2^ (CUMS) = 0.9721, R^2^ (CUMS+XYS1) = 0.5209. (D) Linear correlation analysis of complement C3 with Bacteroidota in mouse gut microbiota; R^2^ (CUMS) = 0.4099, R^2^ (CUMS+XYS1) = 0.7305.

## Data Availability

The data that support the findings of this study are available from the corresponding author upon reasonable request.
