# Supplementary figures and images for: Panacis Quinquefolii Radix Polysaccharides Alleviate Depressive‐Like Behaviors in Chronic Unpredictable Mild Stress‐Induced Mice by Suppressing Complement C1Q/C3‐Mediated Microglial Synaptic Pruning and Modulating Gut Microbiota
Source: CNS Neurosci Ther. 2026 Mar 31;32(4):e70859. doi: 10.1002/cns.70859 (PMC13140852; doi:10.1002/cns.70859)

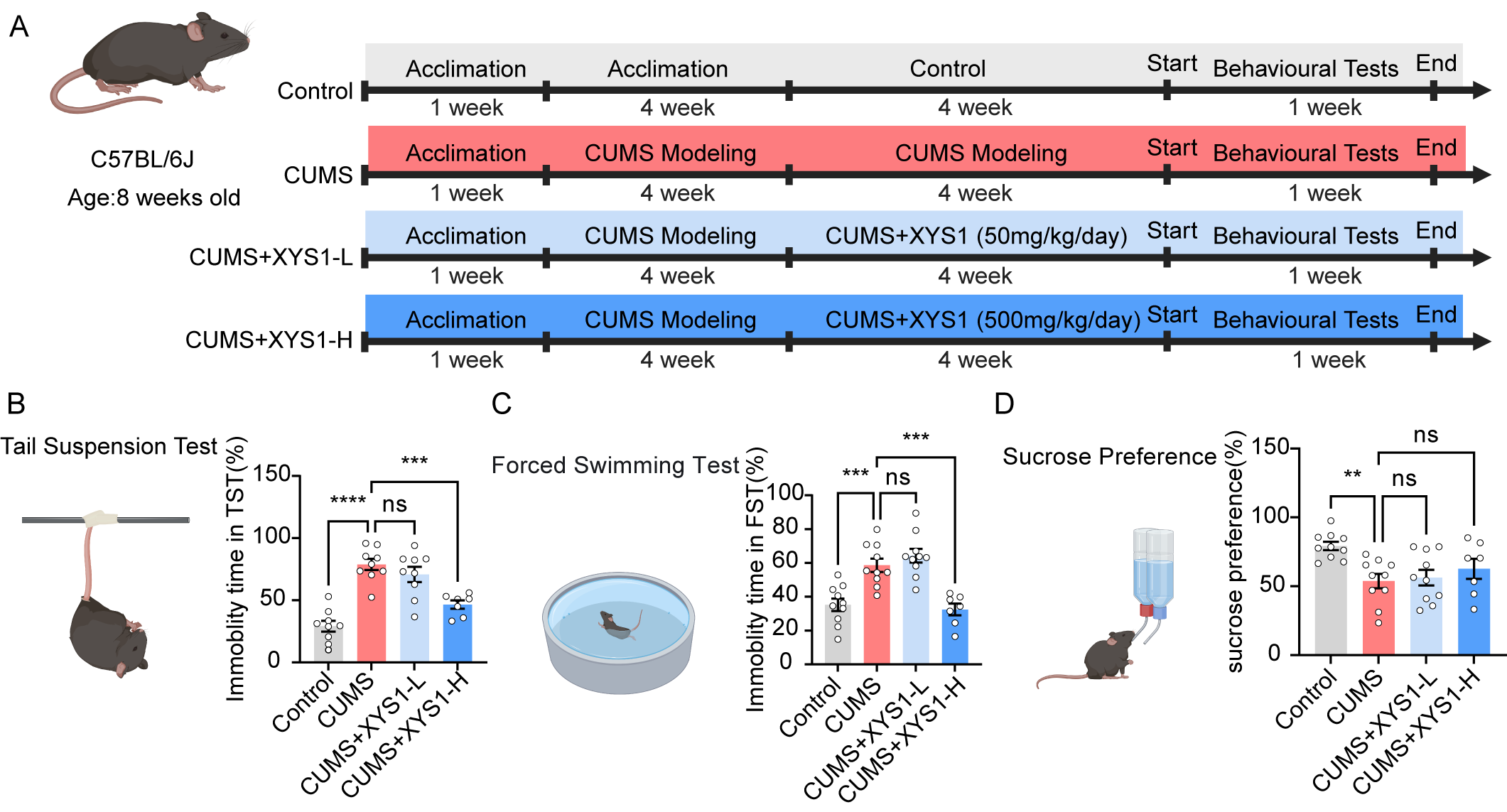

Supplement: Supplementary file 1 — Figure S1: XYS1 relieved CUMS‐induced depressive behavior in a dose‐dependent manner. (A) Experimental timeline of administration of XYS1‐L and XYS1‐H in CUMS mice. Created with Biorender.com. (B, C) Immobility time in the tail suspension test (TST) (B) and the forced swimming test (FST) (C). (D) Quantification of sugar preference in the sucrose preference test (SPT). Data are represented as mean ± SEM. One‐way ANOVA with Dunnett's multiple comparisons test for (B‐D) (n ≥ 7 per group, data of XYS1‐H is related to Figure 1F–H), compared to CUMS group. Statistical significance as follows: ns (not significant), **p < 0.01, ***p < 0.001, ****p < 0.0001. [file CNS-32-e70859-s004.tif]

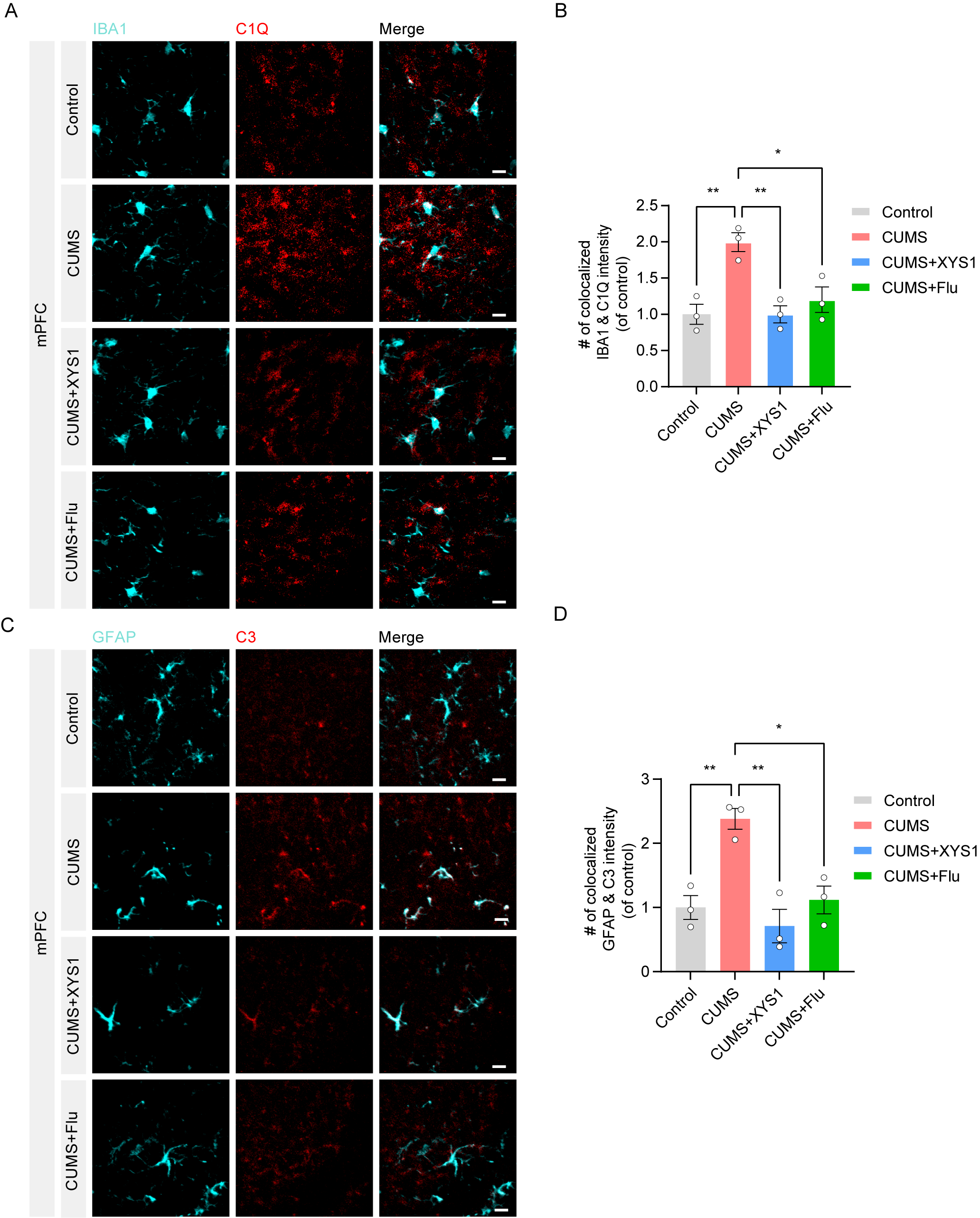

Supplement: Supplementary file 2 — Figure S2: XYS1 alleviated CUMS‐induced increase of complement C1Q and C3 in the mPFC. (A, B) Immunofluorescence staining and quantification of IBA1‐labeled microglia (cyan) and C1Q (red) co‐localized in the mPFC. Scale bar = 10 μm. (C, D) Immunofluorescence staining of GFAP‐labeled astrocyte (cyan) and C3 (red) co‐stained in the mPFC. Scale bar = 10 μm. Data are represented as mean ± SEM. One‐way ANOVA with Tukey's multiple comparisons test for (B and D) (n = 3 per group). Statistical significance as follows: ns (not significant), *p < 0.05, **p < 0.01. [file CNS-32-e70859-s005.tif]

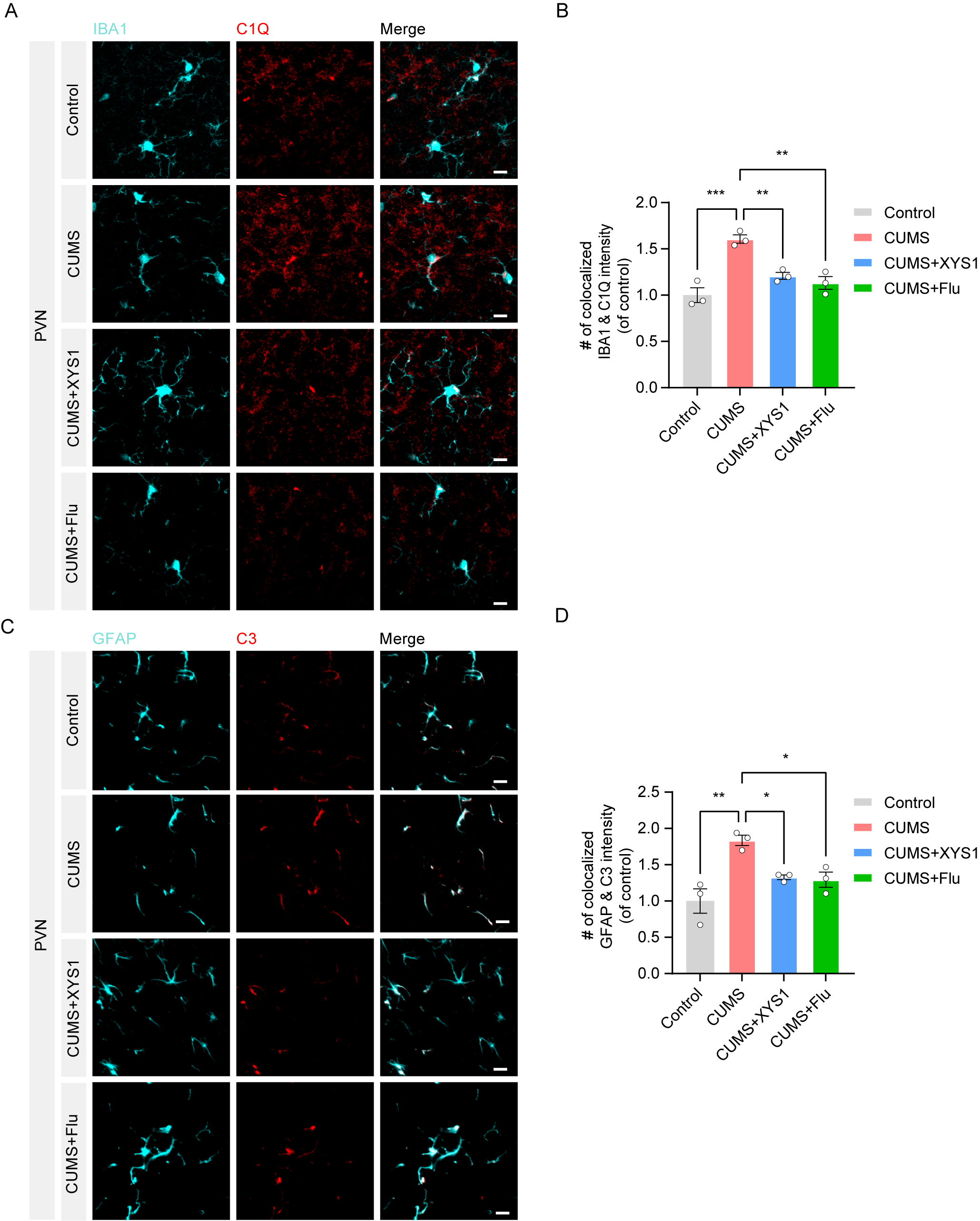

Supplement: Supplementary file 3 — Figure S3: XYS1 alleviated CUMS‐induced increase of complement C1Q and C3 in the PVN. (A, B) Immunofluorescence staining and quantification of IBA1‐labeled microglia (cyan) and C1Q (red) co‐localized in the PVN. Scale bar = 10 μm. (C, D) Immunofluorescence staining of GFAP‐labeled astrocyte (cyan) and C3 (red) co‐stained in the PVN. Scale bar = 10 μm. Data are represented as mean ± SEM. One‐way ANOVA with Tukey's multiple comparisons test for (B and D) (n = 3 per group). Statistical significance as follows: ns (not significant), *p < 0.05, **p < 0.01, ***p < 0.001. [file CNS-32-e70859-s002.tif]

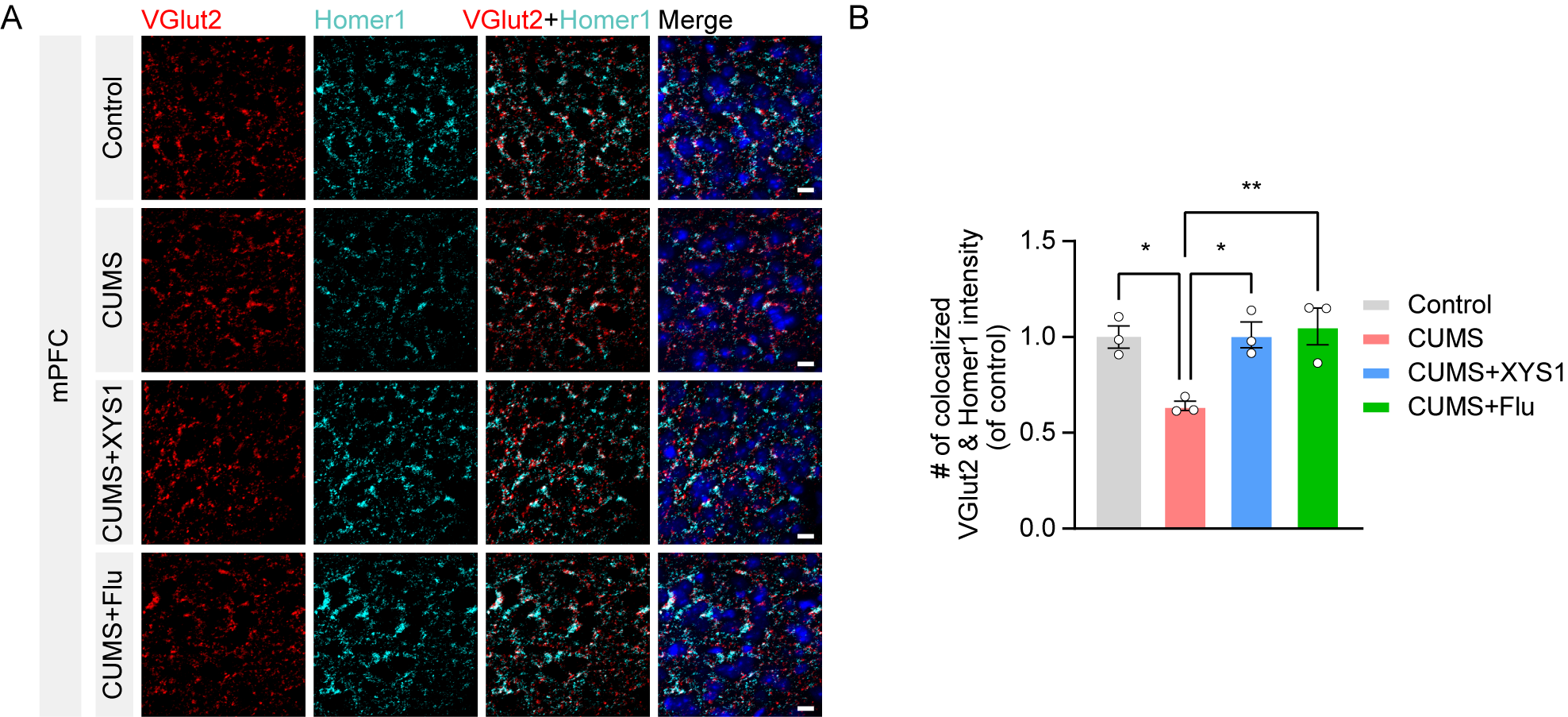

Supplement: Supplementary file 4 — Figure S4: XYS1 ameliorated CUMS‐induced excitatory synapse reduction in the mPFC. (A) Immunofluorescence staining of VGlut2/Homer1 synaptic density in the mPFC. Representative images showing co‐localized VGlut2 (red) and Homer1 (cyan), with nuclei labeled by DAPI (blue). Scale bar = 10 μm. (B) Quantification of colocalization of VGlut2 and Homer1 in the mPFC region for (A). Data are represented as mean ± SEM. One‐way ANOVA with Tukey's multiple comparisons test for (B) (n = 3 per group). Statistical significance as follows: ns (not significant), *p < 0.05, **p < 0.01. [file CNS-32-e70859-s003.tif]

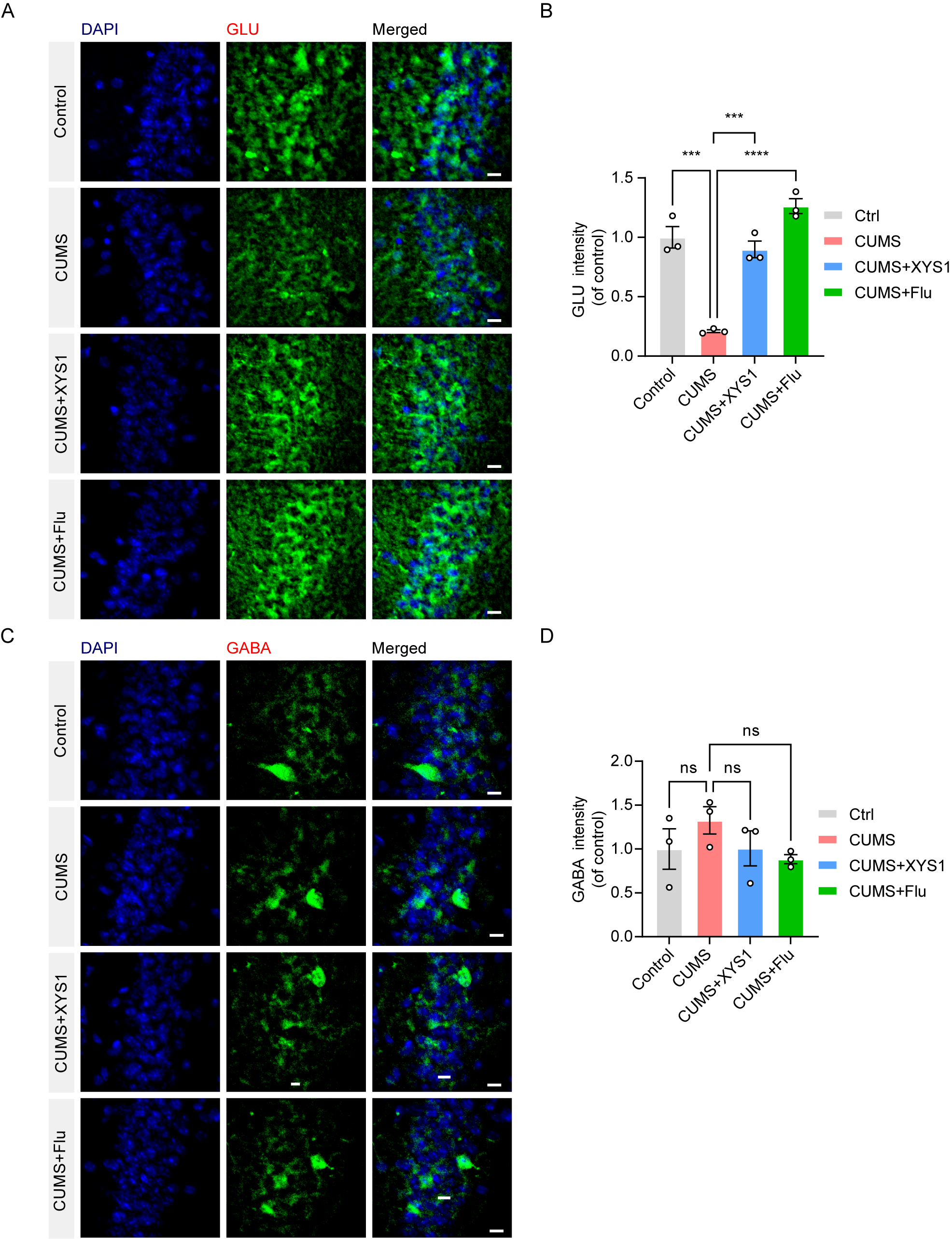

Supplement: Supplementary file 5 — Figure S5: XYS1 attenuated CUMS‐induced reduction in glutamatergic neurons in the hippocampus. (A, B) Immunofluorescence staining and quantification of glutamatergic neurons (Glu, green) in the hippocampus. Scale bar = 10 μm. (C, D) Immunofluorescence staining of GABAergic neurons (GABA, green) in the hippocampus. Scale bar = 10 μm. Data are represented as mean ± SEM. One‐way ANOVA with Tukey's multiple comparisons test for (B and D) (n = 3 per group). Statistical significance as follows: ns (not significant), ***p < 0.001, ****p < 0.0001. [file CNS-32-e70859-s001.tif]

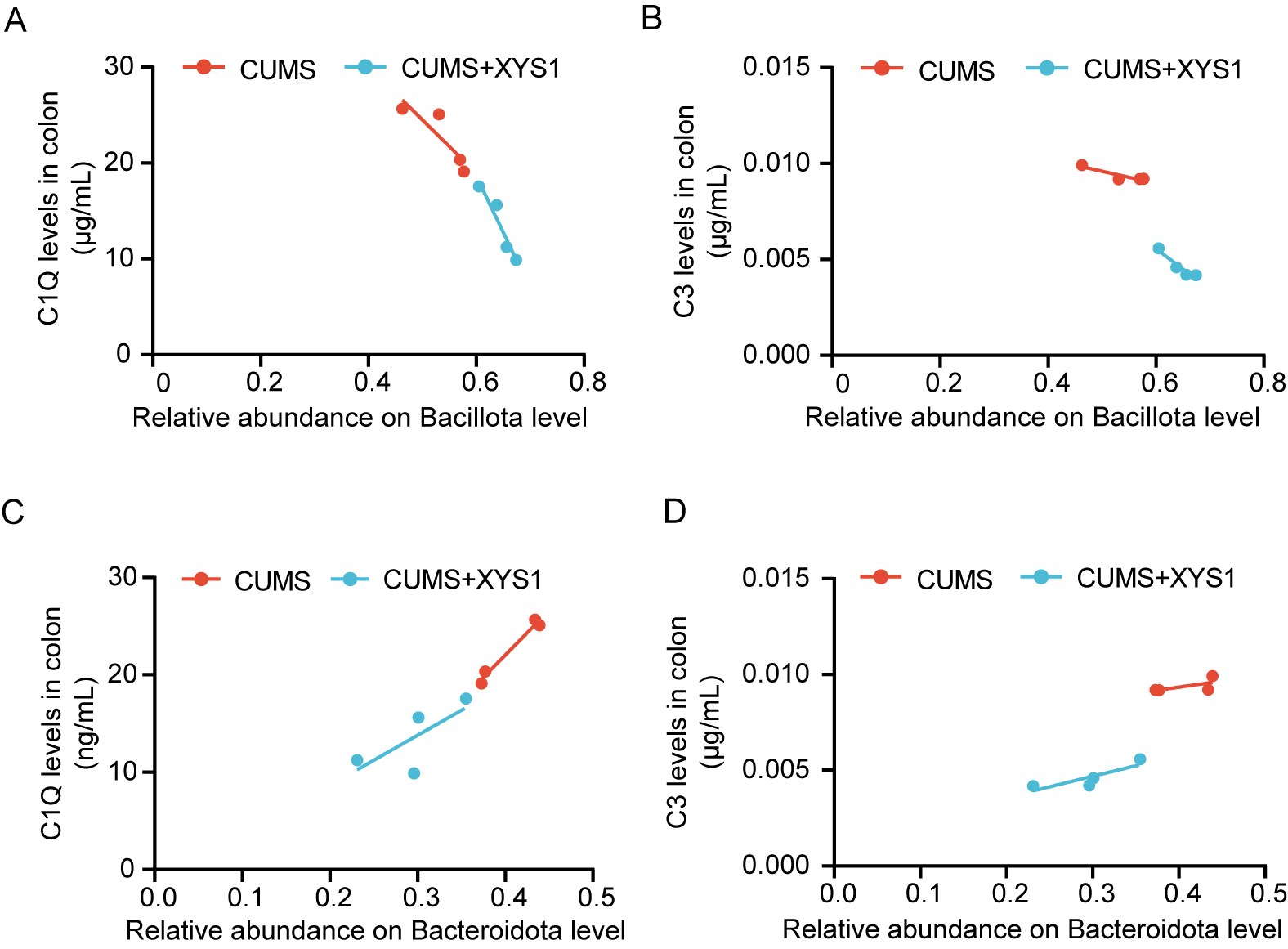

Supplement: Supplementary file 6 — Figure S6: Correlation analysis of key gut microbial phyla with colonic complement C1Q and C3 levels in CUMS mice after XYS1 treatment. (A) Linear correlation analysis of complement C1Q with Bacillota in mouse gut microbiota; R2 (CUMS) = 0.7757, R2 (CUMS+XYS1) = 0.9244. (B) Linear correlation analysis of complement C3 with Bacillota in mouse gut microbiota; R2 (CUMS) = 0.8334, R2 (CUMS+XYS1) = 0.9188. (C) Linear correlation analysis of complement C1Q with Bacteroidota in mouse gut microbiota; R2 (CUMS) = 0.9721, R2 (CUMS+XYS1) = 0.5209. (D) Linear correlation analysis of complement C3 with Bacteroidota in mouse gut microbiota; R2 (CUMS) = 0.4099, R2 (CUMS+XYS1) = 0.7305. [file CNS-32-e70859-s006.tif]
